# Molecular Characterization of the ClpC AAA+ ATPase in the Biology of Chlamydia trachomatis

**DOI:** 10.1128/mbio.00075-23

**Published:** 2023-03-28

**Authors:** Stefan Pan, Aaron A. Jensen, Nicholas A. Wood, Beate Henrichfreise, Heike Brötz-Oesterhelt, Derek J. Fisher, Peter Sass, Scot P. Ouellette

**Affiliations:** a Department of Microbial Bioactive Compounds, Interfaculty Institute of Microbiology and Infection Medicine, University of Tübingen, Tübingen, Germany; b Department of Pathology and Microbiology, College of Medicine, University of Nebraska Medical Center, Omaha, Nebraska, USA; c Institute for Pharmaceutical Microbiology, University of Bonn, Bonn, Germany; d Cluster of Excellence-Controlling Microbes to Fight Infections, Tübingen, Germany; e School of Biological Sciences, Southern Illinois University Carbondale, Carbondale, Illinois, USA; University of Maryland, Baltimore; University of Maryland School of Medicine

**Keywords:** Chlamydia, ClpC, ClpP, AAA+ ATPase, Clp protease, differentiation, development

## Abstract

Bacterial AAA+ unfoldases are crucial for bacterial physiology by recognizing specific substrates and, typically, unfolding them for degradation by a proteolytic component. The caseinolytic protease (Clp) system is one example where a hexameric unfoldase (e.g., ClpC) interacts with the tetradecameric proteolytic core ClpP. Unfoldases can have both ClpP-dependent and ClpP-independent roles in protein homeostasis, development, virulence, and cell differentiation. ClpC is an unfoldase predominantly found in Gram-positive bacteria and mycobacteria. Intriguingly, the obligate intracellular Gram-negative pathogen Chlamydia, an organism with a highly reduced genome, also encodes a ClpC ortholog, implying an important function for ClpC in chlamydial physiology. Here, we used a combination of *in vitro* and cell culture approaches to gain insight into the function of chlamydial ClpC. ClpC exhibits intrinsic ATPase and chaperone activities, with a primary role for the Walker B motif in the first nucleotide binding domain (NBD1). Furthermore, ClpC binds ClpP1P2 complexes via ClpP2 to form the functional protease ClpCP2P1 *in vitro*, which degraded arginine-phosphorylated β-casein. Cell culture experiments confirmed that higher order complexes of ClpC are present in chlamydial cells. Importantly, these data further revealed severe negative effects of both overexpression and depletion of ClpC in Chlamydia as revealed by a significant reduction in chlamydial growth. Here, again, NBD1 was critical for ClpC function. Hence, we provide the first mechanistic insight into the molecular and cellular function of chlamydial ClpC, which supports its essentiality in Chlamydia. ClpC is, therefore, a potential novel target for the development of antichlamydial agents.

## INTRODUCTION

Proteostasis in any organism is a complex process requiring the careful coordination of regulatory factors acting at each level of gene expression and protein regulation. In bacteria, the caseinolytic protease (Clp) is known to contribute to regulated proteolysis and has been well studied in this regard (1–4). Clp is a highly conserved macromolecular protease comprised of two functionally distinct components: a tetradecameric proteolytic ClpP core and a corresponding hexameric type I AAA+ (ATPases associated with diverse cellular activities) unfoldase such as ClpX or ClpC (5). Regulated protein hydrolysis requires the interaction of ATP-fueled AAA+ unfoldases with the ClpP core. The unfoldase recognizes, unfolds, and translocates protein substrates into the degradation chamber of the ClpP core (5, 6). Importantly, AAA+ unfoldases also function as protein chaperones independently of ClpP (7–10) and thus are crucial for bacterial physiology via multiple routes. These include both ClpP-dependent and ClpP-independent roles in protein homeostasis, development, virulence, cell differentiation, genetic competence, and, in some instances, viability (11–15). Unsurprisingly, in recent years, ClpP and AAA+ unfoldases have emerged as promising target structures for drug development (16–24).

The Clp protease is conserved within the obligate intracellular pathogen Chlamydia trachomatis (Ctr), the world’s leading cause of preventable infectious blindness and bacterial sexually transmitted infections. Chlamydia species are Gram-negative bacteria that undergo a unique and complex biphasic developmental cycle. Infection of a susceptible host cell begins with the internalization of the small, electron dense, and nondividing form of Chlamydia called the elementary body (EB) (25, 26). Once internalized into a host-derived vacuole termed the inclusion, the EB immediately begins a primary differentiation event into the larger, less electron dense, noninfectious replicating form termed the reticulate body (RB). The first cell division event is followed by rapid multiplication of RBs by an asymmetric polarized cell division mechanism (27, 28). Secondary differentiation proceeds asynchronously as RBs condense into EBs (27). These EBs will then exit the host cell through host cell lysis or extrusion of the inclusion itself. Given the distinct functional, morphologic, and proteomic differences between EBs and RBs, as well as the processes of differentiation that generate them, we have hypothesized that regulated protein turnover is a critical and essential aspect of Chlamydia physiology and pathogenesis. We have further hypothesized and presented evidence that the chlamydial Clp system is a key mediator of differentiation in these organisms (29–31).

In evolving to obligate intracellular dependence, Chlamydia species have significantly reduced their genome size and content, and this is notable for Ctr, which encodes roughly 900 open reading frames (ORFs) in ~1 Mbp. This suggests that the presence of a given gene in the genome indicates an important, if not essential, function. Of interest, Ctr possesses two *clpP* genes (*clpP1* and *clpP2*). Of further interest is the presence of two AAA+ unfoldases encoded within the chlamydial genome: *clpX* and *clpC*. The *clpP2* and *clpX* genes are organized within an operon while *clpC* and *clpP1* are positioned separately on the chromosome (31, 32). That Chlamydia should maintain two copies of a gene in its chromosome suggests an important function, especially since bacteria with two or more *clpP* genes are comparatively rare. We previously investigated the ClpX and ClpP protease components of Ctr *in vitro* (30, 32), revealing the presence of an atypical, mixed ClpP core containing both ClpP1 and ClpP2. Combined with the AAA+ unfoldase ClpX, the chlamydial ClpXP complex displayed robust proteolytic activity for model substrates both in cell culture (30) and *in vitro* (30, 32) experiments. More recently, we demonstrated a critical function of chlamydial ClpX in mediating secondary differentiation via changes in its substrate preferences between SsrA-tagged and untagged substrates (29). We have further demonstrated that overexpressing catalytically inactive, but not wild-type, mutants of ClpP1, ClpP2, or ClpX in Ctr has a negative impact on chlamydial growth and development (31). These data support our hypothesis that the Clp system is critical for developmental cycle progression in Chlamydia.

While ClpX is well conserved across all types of eubacteria and even mitochondria, ClpC is typically found in Gram-positive bacteria and mycobacteria. ClpC has been shown to recognize substrates containing a phosphorylated arginine residue and is associated with the type III heat shock response in Bacillus subtilis (33). Given the presence of this second AAA+ unfoldase, not typically found in Gram-negative bacteria, we hypothesized that ClpC may serve a specialized and essential function in Chlamydia. The current study was designed to characterize the *in vitro* enzymatic activity of ClpC and its putative interplay with ClpP in regulated proteolysis as well as the effects of dysregulating ClpC expression in Chlamydia within cell culture. Multiple sequence alignments of chlamydial ClpC between heterologous species demonstrated that chlamydial orthologs possess the critical residues necessary for activity, including two nucleotide binding domains (NBD1/2). Principle findings from our study indicate that chlamydial ClpC functions as an ATPase capable of delivering a substrate to the ClpP1P2 protease core for degradation. Further, we demonstrated that dysregulating ClpC expression through exogenous overexpression or knockdown of *clpC* transcripts has detrimental effects on chlamydial growth. Overall, our efforts have expanded our understanding of the function of the Clp protease system in Chlamydia by revealing that the activity of ClpC is essential for normal developmental cycle progression.

## RESULTS

### Chlamydial ClpC retains the conserved motifs essential for ClpC activity.

**FIG 1:**
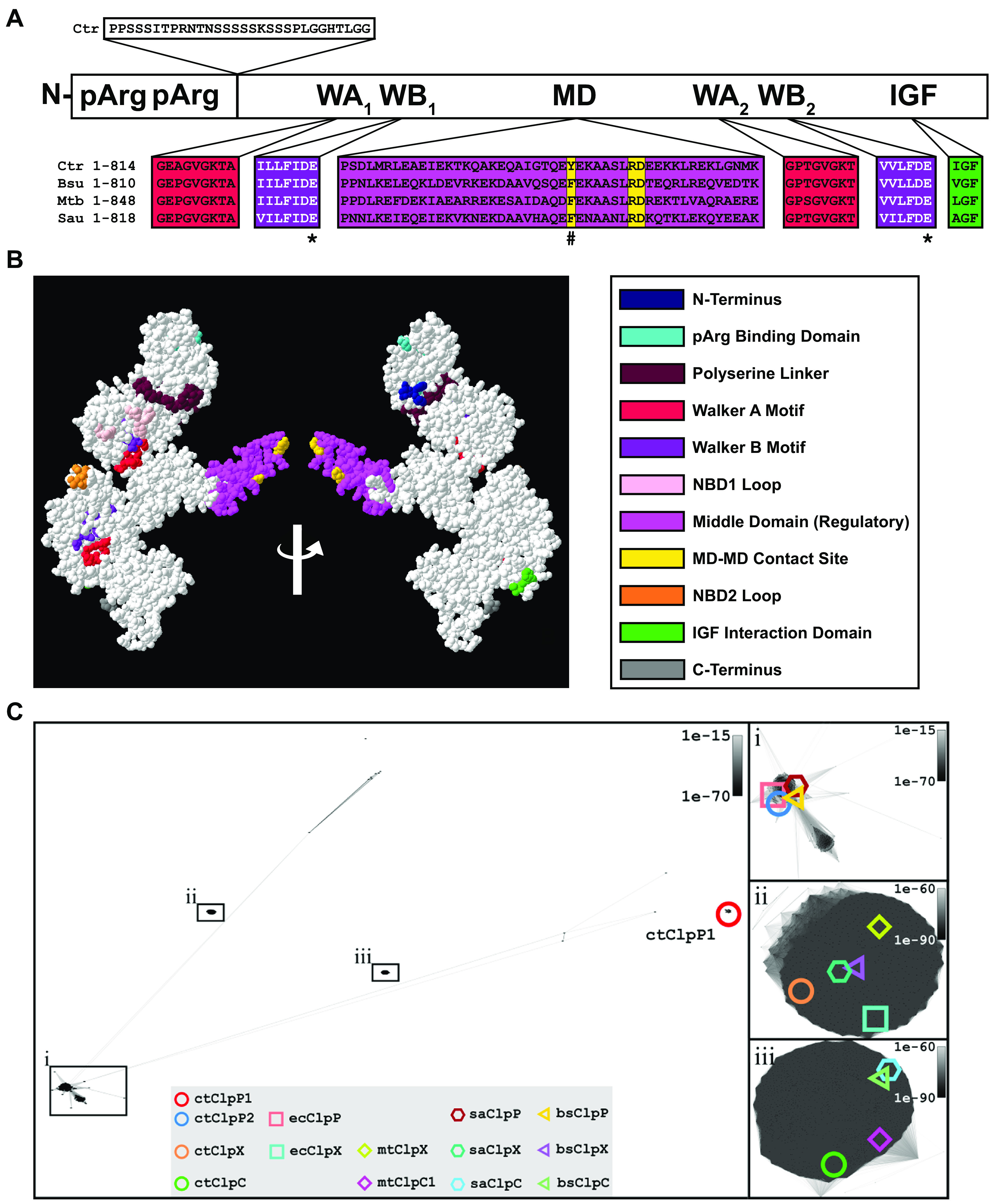
Chlamydial ClpC retains conserved functional domains and clusters with ClpC orthologs. (A) Multiple sequence alignment of ClpC protein sequences between Gram-negative C. trachomatis (Bu 434) and Gram-positive B. subtilis (strain 168), M. tuberculosis (H37Rv), and S. aureus (PS47). Color code is shown below (WA, walker A motif; WB, walker B motif; NBD1 or NBD2, nucleotide binding domain 1 or 2, respectively; MD, middle-domain contact site for oligomerization; IGF interaction domain, region of interaction with ClpP binding cleft). Asterisks indicate Walker B sites that were mutated from glutamic acid to alanine in this study to remove catalytic activity of NBDs. The nonconserved tyrosine within the MD-MD contact site is denoted by the hashtag symbol. (B) 3D color-coded modeling of a ClpC monomer viewed from each face. (C) Cluster analyses of ClpP, ClpX, and ClpC. Distance plots based on CLANS (39). *P* value threshold was set to 1e-75. (i) Enlarged view of the major ClpP clusters. (ii) Enlarged view of the ClpX cluster. (iii) Enlarged view of the ClpC cluster.

To examine if Ctr ClpC has the expected sequence elements to function as a class I AAA+ unfoldase, we took a bioinformatics approach by aligning its primary amino acid sequence to those of well-studied ClpC orthologs from model bacteria. Conserved regions and functional motifs are indicated in Fig. 1A (see Fig. S1 for full-length alignment). The conserved Walker-type nucleotide binding domains (NBDs) represent hallmark features of the Hsp100 protein family and are required for ATPase activity (34, 35). Unlike ClpX, an Hsp100 chaperone with a single NBD (i.e., class II), ClpC contains two highly conserved NBDs (NBD1 and NBD2; i.e., class I). The two canonical Walker A/B motifs of the NBDs characteristic of ClpC proteins are highly conserved between orthologous proteins, suggesting ATPase function is conserved in the chlamydial protein (red, purple). Importantly, the critical glutamate residues involved in ATP hydrolysis are present in the Walker B motifs (E306/E644 in the chlamydial protein) (36). The middle domain contact sites (yellow) necessary for oligomerization are present, albeit with an alteration in the chlamydial ortholog from phenylalanine 462 to tyrosine (F462Y). The IGF domain (green), important for interactions with ClpP, is also present. The N-terminal domain involved in the recognition of phosphorylated arginine (pArg) and other specific substrates and adaptors (37, 38) is present in the chlamydial ClpC, but, perhaps not surprisingly given the likely substrate differences between species, shows more sequence variability beyond the conserved pArg recognition residues. Unique to the chlamydial ClpC is an ~20 amino acid serine-rich linker connecting the N-terminal substrate recognition domain to the ATPase domain. Unsurprisingly, the different ClpC orthologs in the different chlamydial serovars and species were highly conserved (Fig. S2).

10.1128/mbio.00075-23.1 FIG S1 Chlamydial ClpC retains conserved functional domains compared to other orthologs. Multiple sequence alignment of ClpC protein sequences between Gram-negative C. trachomatis (Bu 434) and Gram-positive B. subtilis (strain 168), M. tuberculosis (H37Rv), and S. aureus (PS47). Color code is shown below (WA, walker A motif, WB, walker B motif; NBD1 or NBD2, nucleotide binding domain 1 or 2, respectively; MD, middle-domain contact site for oligomerization; IGF interaction domain, region of interaction with ClpP binding cleft). To remove catalytic activity, Walker B sites were mutated from glutamic acid to alanine denoted by *. The nonconserved tyrosine within the MD-MD contact site is denoted by #. Download FIG S1, PNG file, 1.6 MB. Copyright © 2023 Pan et al. 2023 Pan et al. https://creativecommons.org/licenses/by/4.0/ This content is distributed under the terms of the Creative Commons Attribution 4.0 International license.

10.1128/mbio.00075-23.2 FIG S2 Chlamydial ClpC retains conserved functional domains between serovars and species. Multiple sequence alignment of ClpC protein sequences between human-pathogenic C. trachomatis: serovar A (HAR-13), serovar D (UW-3/CX), serovar L2 (Bu 434); other human chlamydial species: Chlamydia psittaci (6BC genotype A) and Chlamydia pneumoniae (AR39); chlamydial species that infect other species: Chlamydia suis (infects pigs), Chlamydia muridarum (infects mice); and distantly related parachlamydia: Parachlamydia acanthamoebae (infects amoeba). Glutamic acid to alanine mutations in Walker B sites are denoted by *. The non-conserved tyrosine within the MD-MD contact site between chlamydial ClpC orthologs and other Gram-positive bacteria is denoted by #. Download FIG S2, PNG file, 1.0 MB. Copyright © 2023 Pan et al. 2023 Pan et al. https://creativecommons.org/licenses/by/4.0/ This content is distributed under the terms of the Creative Commons Attribution 4.0 International license.

We subsequently modeled chlamydial ClpC using a protein data bank from Phyre2 and 3D-rendered using SWISS-MODEL software. As seen in Fig. 1B, the three-dimensional (3D) model of ClpC shows the Walker A/B motifs within close proximity to bind and hydrolyze ATP. The IGF domain is located at a surface exposed region consistent with where ClpP would bind. Overall, these data indicate that the chlamydial ClpC ortholog has all the sequence elements required for a bona fide ClpC ATPase.

### Clustering analysis indicates chlamydial ClpC and ClpX are highly conserved across species.

In homology relationship analyses, we previously showed that the ClpP isoforms of Ctr are nonparalogous, as both ClpP1 and ClpP2 were located at distinct and separate subgroups (32). In the current study, we extended our analysis by including the amino acid sequences of the AAA+ unfoldases ClpX and ClpC into a homology relationship plot based on Cluster Analysis of Sequences (CLANS) (32, 39), thereby obtaining a comprehensive picture of homology relationships of the entire chlamydial Clp system. In addition to 597 unique ClpP sequences from the UniProtKB/Swiss-Prot database, 493 unique ClpX sequences from the UniProtKB/Swiss-Prot database, and 1,671 unique ClpC sequences (34 from UniProtKB/Swiss-Prot and 1,637 from UniProtKB/TrEMBL database) were used to generate a cluster map based on all-against-all pairwise sequence similarities (Fig. 1C). In agreement with our previous results, ClpP1 and ClpP2 are spatially separated from each other, despite their apparent interdependence on a functional level (31, 32). For chlamydial ClpX and ClpC, respectively, nearly all sequences appeared to form one single homogenous cluster and, in contrast to ClpP, no distinct subcluster formation was computable. Also, the highly conserved (L/I/V)-G-(F/L) ClpP-binding motif, which exists in both chlamydial ClpC and ClpX, was found in 84% of all ClpC and 89% of all ClpX sequences despite high sequence variability in nonconserved regions (Fig. S3). Our data thus indicate a close paralogous relationship between ClpX/C orthologs and a highly conserved local protein domain architecture.

10.1128/mbio.00075-23.3 FIG S3 Conservation of the ClpP-binding motif in ClpC and ClpX. Typical variants of the ClpP-binding motif (L/I/V)-G-(F/L) were found in 84% of all ClpC and 89% of all ClpX sequences. Partially conserved binding motifs such as (A/F/M/T/Y)-GF are found in roughly 10% of each group of Clp ATPases. Remaining sequences (6% for ClpC, 0.6% for ClpX) showed no apparent conservation. Download FIG S3, PNG file, 0.3 MB. Copyright © 2023 Pan et al. 2023 Pan et al. https://creativecommons.org/licenses/by/4.0/ This content is distributed under the terms of the Creative Commons Attribution 4.0 International license.

### The Walker B motif of ClpC NBD1 disproportionally contributes to ATPase and chaperone activities.

**FIG 2:**
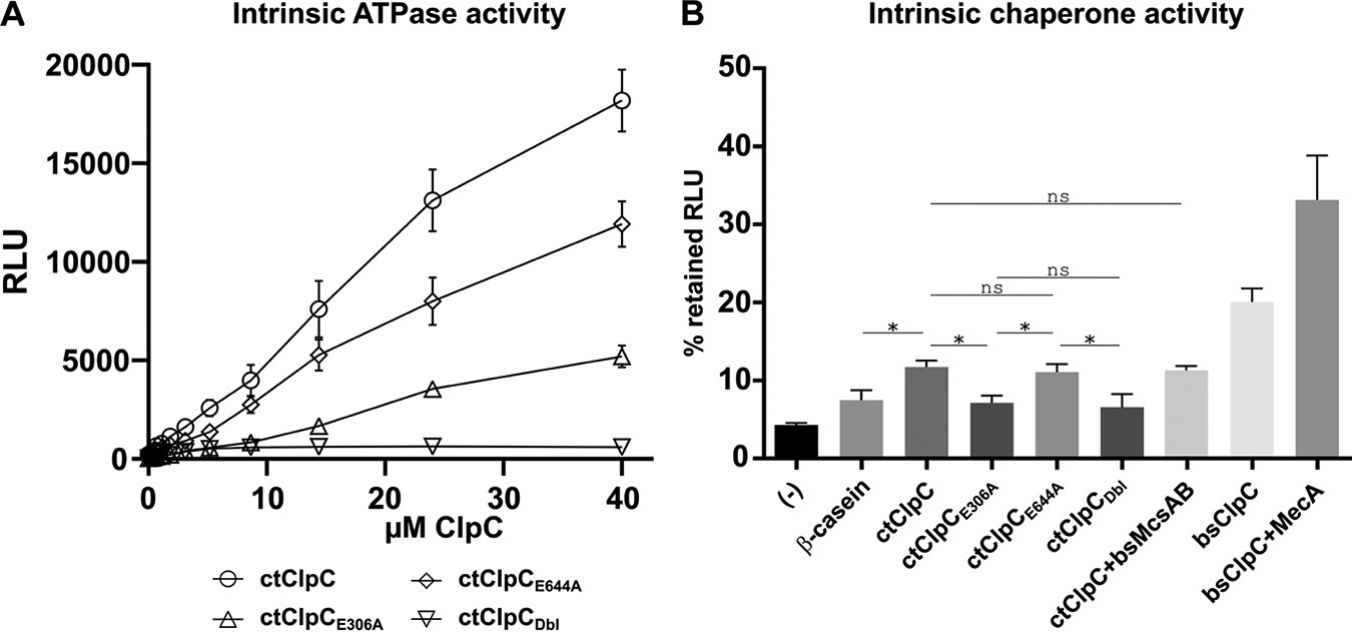
The Walker B motif of Ctr ClpC NBD1 disproportionally contributes to ATPase and chaperone activities. (A) Intrinsic ATPase activity of wild-type and mutant ClpC using a quantitative ADP to ATP system followed by luciferase activity readout. (B) Intrinsic chaperone activity of wild-type and mutant ClpC in a protein aggregation prevention assay. The residual activity of heat-treated luciferase was determined in the absence or presence of either β-casein (nonchaperone control assay) or ClpC from C. trachomatis (ct) or B. subtilis (bs) as indicated. β-casein was used as reference protein to assess nonspecific protein-stabilizing effects. The statistical significance analysis was performed via two-way ANOVA determination (*, *P* < 0.05; ns, not significant). As a positive control, chaperone activity was also determined for bsClpC, which was substantial and could be further elevated by the adaptor protein MecA.

To begin characterizing chlamydial ClpC, we investigated its ATPase activity *in vitro* using recombinant protein preparations. Using a quantitative ADP detection system coupled to luciferase activity readout, wild-type ClpC exhibited ATPase activity in a dose-dependent manner (Fig. 2A). Furthermore, inactivation of the Walker B motif in NBD1, conferred by introducing an E306A mutation, decreased hydrolysis activity more strongly than inactivation of the Walker B motif in NBD2 (E644A). While inactivation of NBD2 reduced ATPase activity by approximately 30%, mutation of NBD1 reduced overall ATPase activity by over 70%, indicating a dominant role of the NBD1 Walker B motif for ATPase activity of ClpC.

Next, we analyzed the chaperone activity of Ctr ClpC in a protein aggregation prevention assay using heat-induced denaturation of luciferase. Compared to the control assay using β-casein, the addition of wild-type ClpC from either Ctr or B. subtilis (Bs) increased luciferase activity and thus prevented, to some degree, the heat-induced denaturation of the luciferase protein (Fig. 2B). Corroborating our results for ClpC ATPase activity, the Walker B motifs in NBD1 and NBD2 displayed an unequal contribution to the chaperone activity. While the E644A mutant showed unaltered chaperone activity compared to wild-type ClpC, the E306A mutant and the double mutant Dbl (E306A, E644A) did not prevent luciferase aggregation above the β-casein control, thereby highlighting the importance of the Walker B motif in NBD1 for ClpC activity. Addition of β-casein led to a low level of stabilization of the luciferase as indicated by an increase of luciferase activity above background where neither β-casein nor wild-type ClpC was added. This suggests some nonspecific stabilizing effects by proteins with nonchaperone functions, which was also observed for the E306A and Dbl mutant of ClpC. ClpC recognizes arginine-phosphorylated substrates. However, the addition of B. subtilis McsAB kinase (bsMcsAB), which confers arginine phosphorylation of substrates, did not enhance Ctr ClpC chaperone activity, suggesting that either luciferase is not arginine-phosphorylated by bsMcsAB in this assay, or arginine phosphorylation may not be a prerequisite for protein disaggregation via ClpC under the conditions tested.

### ClpC binds to the heteromeric ClpP1P2 complex via ClpP2 to form the functional protease ClpCP2P1.

**FIG 3:**
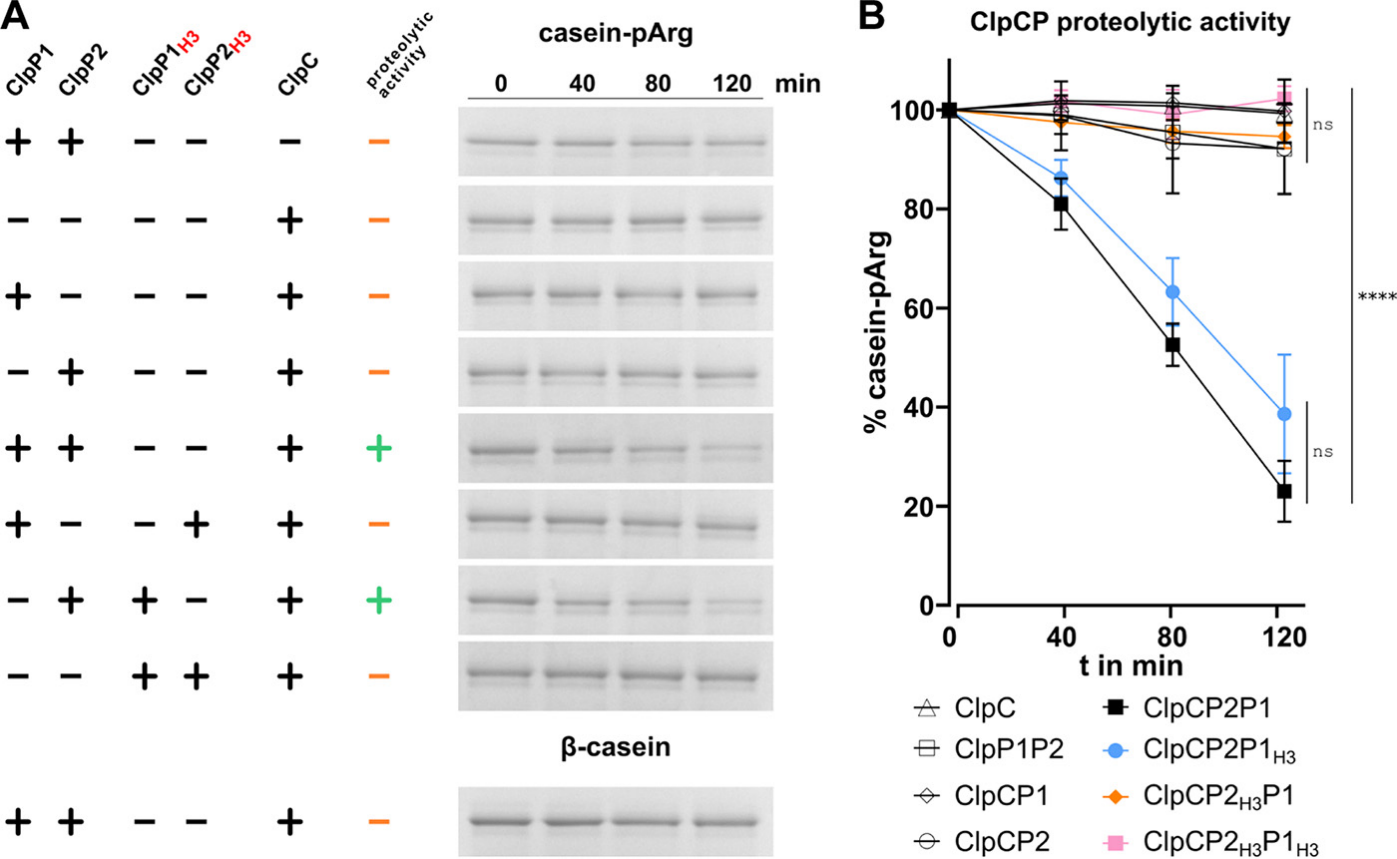
Ctr ClpC binds to the heteromeric ClpP1P2 complex via ClpP2 to form the functional protease ClpCP2P1. (A) Protease activity as determined by degradation of arginine-phosphorylated β-casein (casein-pArg) as a substrate and assessed by SDS-PAGE at 0, 40, 80, and 120 min. Unsuccessful or successful proteolytic activity with each set of proteins are further marked by minus (orange) or plus (green) signs, respectively. ClpP1_H3_ and ClpP2_H3_ mutant proteins carry triple amino acid exchanges to assess the binding of ClpC. Degradation of casein-pArg was only observed in the presence of ClpC, ClpP1, and wild-type ClpP2. Of note, unphosphorylated β-casein was not degraded in the presence of ClpCP2P1, indicating the requirement of arginine phosphorylation for ClpC-mediated degradation. Full SDS-PAGE images, including input controls are provided in Fig. S5. (B) Densitometry of bands shown in panel A. Experiments were performed in triplicate, mean values are presented. The statistical significance analysis was performed via two-way ANOVA determination (****, *P* < 0.0001; ns, not significant).

Due to their size, folded polypeptides and proteins are unable to access the proteolytic chamber and be degraded by ClpP alone (5, 40–43). For proteolytic activity, the ClpP core interacts with associated unfoldases, such as ClpC or ClpX, which unfold protein substrates in an ATP-dependent manner and then feed the unfolded substrates into the ClpP degradation chamber. ClpC recognizes arginine-phosphorylated substrates. Therefore, to assess whether Ctr ClpC could activate ClpP protease activity *in vitro*, we first prepared arginine-phosphorylated β-casein (casein-pArg) as a protein substrate for ClpCP (37, 44). Successful phosphorylation of β-casein was confirmed by pArg-specific antibodies (Fig. S4). In subsequent protease activity assays, we then analyzed the proteolytic capacity of different combinations of ClpC, ClpP1, and ClpP2 to digest casein-pArg (Fig. 3 and Fig. S5). Here, ClpP1P2 did not notably digest casein-pArg in the absence of ClpC. Similarly, ClpC alone did not affect the abundance of casein-pArg. Also, incubation of either ClpP1 or ClpP2 in the presence of ClpC was not sufficient for proteolytic activity. However, when ClpP1, ClpP2, and ClpC were combined, casein-pArg was clearly degraded. Thus, our data show that, like Ctr ClpX (31), ClpC forms a functional protease with a heteromeric complex ClpP1P2.

10.1128/mbio.00075-23.4 FIG S4 McsB-mediated arginine phosphorylation of β-casein. Dot-blot analysis using anti-pArg-antibodies. Strep-tagged McsAB of B. subtilis (bsMcsAB) or McsB of *G. stearothermophilus* (gsMcsB) were removed prior immunoblotting via reverse Strep purification as indicated by *. β-casein is gradually arginine-phosphorylated by bsMcsAB or gsMcsB. In the reverse purified bsMcsAB fraction, auto-phosphorylation of presumably bsMcsB occurs rapidly. Download FIG S4, PNG file, 0.4 MB. Copyright © 2023 Pan et al. 2023 Pan et al. https://creativecommons.org/licenses/by/4.0/ This content is distributed under the terms of the Creative Commons Attribution 4.0 International license.

10.1128/mbio.00075-23.5 FIG S5 Proteolytic activity of wild-type and mutant Ctr Clp complexes. Protease activity as determined by the degradation of arginine-phosphorylated β-casein (casein-pArg) or nonphosphorylated β-casein (casein) as substrates and assessed using SDS-PAGE at 0, 40, 80, and 120 minutes. Conditions of successful proteolytic activity are indicated in green. ClpP1_H3_ and ClpP2_H3_ mutant proteins carry triple amino acid exchanges to assess the binding of ClpC. Degradation of casein-pArg was only observed in the presence of ClpC, ClpP1 and wild-type ClpP2. Unphosphorylated β-casein was not degraded in the presence of ClpCP2P1, indicating the requirement of arginine phosphorylation for ClpC-mediated degradation. Of note, Strep-tagged ctClpP1 (22.4 kDa) and ctClpP2 (23.3 kDa) were not distinguishable on SDS-PAGE. Excerpts of the SDS-PAGE images are shown in Fig. 3A. SDS-PAGE images are representative of at least three independent experiments. Download FIG S5, PNG file, 0.9 MB. Copyright © 2023 Pan et al. 2023 Pan et al. https://creativecommons.org/licenses/by/4.0/ This content is distributed under the terms of the Creative Commons Attribution 4.0 International license.

We next sought to identify the binding partner of ClpC within the heteromeric ClpP core. Unlike *clpX*, which is included within an operon with *clpP2*, the *clpC* gene is not located closely to either *clpP1* or *clpP2* (31, 32), and thus interaction of ClpC with either ClpP homolog was not predictable based on the genomic context. To characterize ClpC binding to ClpP, we constructed mutants of ClpP1 and ClpP2 with amino acid changes in their respective Clp ATPase binding regions that were based on known homologous ClpP sequences, resulting in ClpP1_H3_ (V57A, M77A, L186T) and ClpP2_H3_ (F63A, F83A, I190T). When we substituted wild-type ClpP1 and/or ClpP2 for the respective mutant proteins in casein-pArg degradation assays, ClpCP2_H3_P1 as well as ClpCP2_H3_P1_H3_ were devoid of proteolytic activity, while casein-pArg degradation was almost fully retained in the ClpCP2P1_H3_ mutant (Fig. 3). Hence, our results show that ClpC binds to the heteromeric ClpP1P2 complex via ClpP2. Of note, instead of residual rates of substrate degradation where approximately 50% is conferred via the wild-type ClpP1/2 protein (32), ClpP1_H3_P2 retained proteolytic activity like wild-type ClpP1P2 for degrading casein-pArg. This was not the case with ClpP1P2_H3_, as no degradation was detected. These data indicate that the H3 mutations in ClpP1_H3_ do not affect ClpP activity whereas the mutations in ClpP2_H3_ prevent proteolytic activity of the complex by disturbing interactions with ClpC. Importantly, we further observed that unphosphorylated β-casein was not degraded by ClpCP2P1, indicating that arginine phosphorylation is required for ClpC-mediated degradation in these assays.

### Wild-type and Walker B mutants of ClpC form homo- and heterotypic oligomers.

A key characteristic of ClpC orthologs necessary for their activity is their ability to form a homohexamer through a trimer of dimers (45). Although we detected *in vitro* enzymatic activity of the recombinant ClpC protein, we wanted to verify that higher order complexes exist in the cellular context. Therefore, to determine whether ClpC oligomerizes, we performed a series of bacterial two-hybrid (BACTH) assays in Escherichia coli as well as cross-linking experiments followed by Western blotting during infection in cell culture with wild-type Ctr serovar L2. We also tested oligomerization of ClpC with mutations in the Walker B motifs (i.e., E306A, E644A, and both mutations [Dbl]) to assess and confirm that these mutations did not impair the ability of ClpC to form oligomers in other analyses.

**FIG 4:**
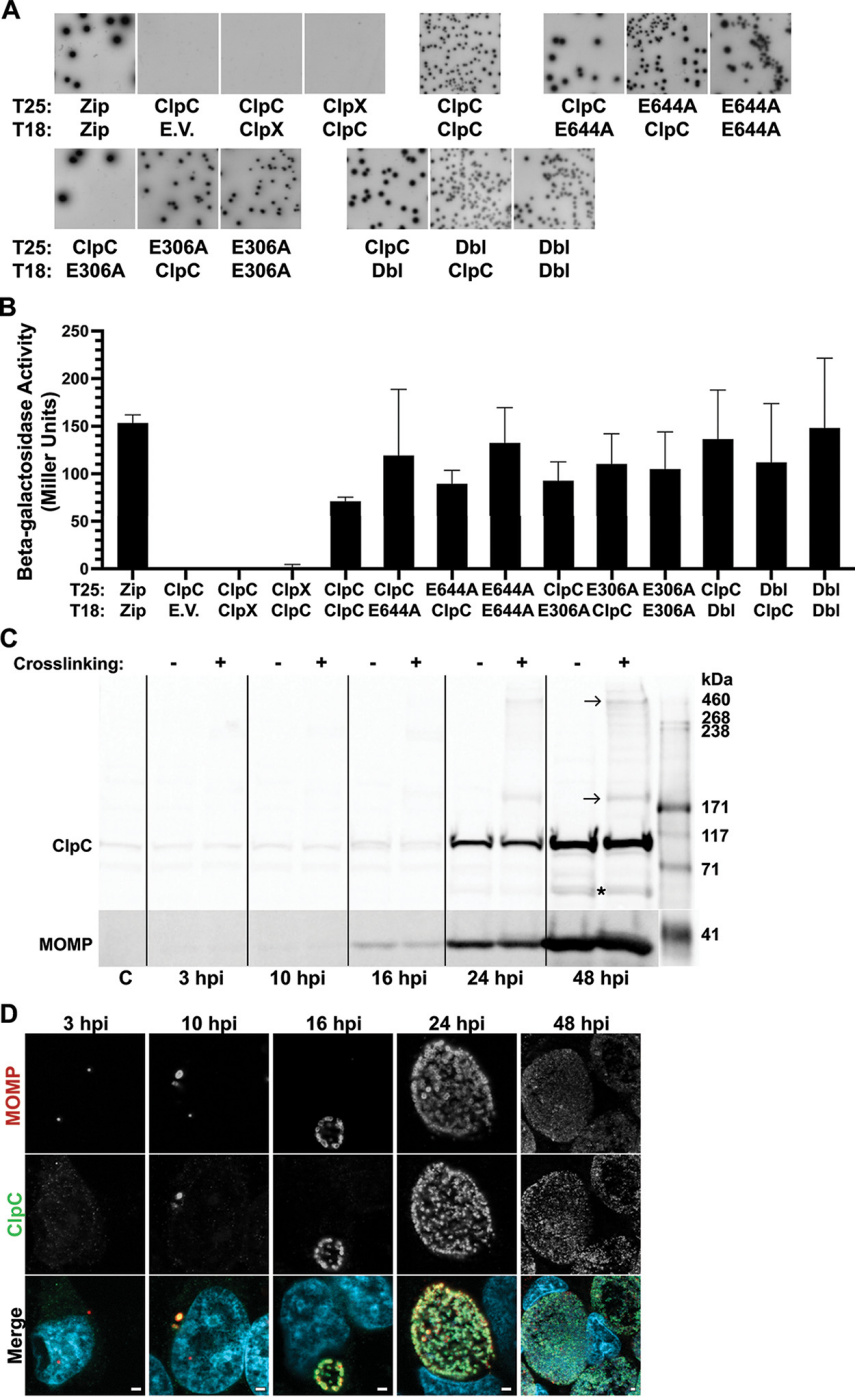
Wild-type and mutant ClpC interact both homotypically and heterotypically. (A) Bacterial adenylate cyclase two-hybrid (BACTH) assays showing homotypic interactions between either wild-type (ClpC) or mutant (E306A, E644A, or Dbl) ClpC, as well as heterotypic interactions between wild-type and mutant ClpC. Positive (Zip:Zip) or negative (T25-ClpC versus T18-empty vector [E.V.], and ClpC versus Ctr ClpX in either orientation) controls are also shown. Photos have been set to grayscale. (B) Quantification of β-galactosidase activity of the BACTH interactions shown in panel A in Miller units. (C) Crosslinking assays to assess endogenous ClpC oligomerization in cell culture experiments. Wild-type Ctr L2 infected HeLa cells were incubated or not at the indicated hours postinfection (hpi) with the primary amine cross-linker DSS and analyzed by immunoblotting using ClpC-specific antibodies. Arrows indicate expected ClpC dimer and hexamer sizes. A putative degradation product of ClpC within protein samples was consistently observed and is denoted by *. Uninfected HeLa cell lysate was included as a negative control and denoted as C. Major outer membrane protein (MOMP)-specific antibodies served as a control to confirm Ctr infection levels. (D) Detection of endogenous ClpC by immunofluorescence in cell culture experiments. Antibody staining of wild-type Ctr L2 samples collected in parallel with panel C, fixed with MeOH at the indicated hpi, and stained for ClpC (green), MOMP (bacteria; red), and DNA (blue). Merged images of all color channels are shown. Representative images were taken in triplicate on a Zeiss Apotome at ×100 magnification with ×5.5 digital zoom for 3 to 24 hpi and ×2.75 digital zoom for 48 hpi. Scale bar = 2 μm.

The BACTH system operates by the reconstitution of adenylate cyclase activity when two catalytic fragments, T25 and T18 of the Bordetella pertussis Cya, are expressed as fusion proteins with proteins of interest and are brought into proximity by the interacting proteins (46). When expressed separately or as fusions with noninteracting proteins, no cAMP is produced. Reconstituted cAMP production activates β-galactosidase expression, which can be qualitatively assessed on 5-bromo-4-chloro-3-indolyl-β-d-galactopyranoside (X-Gal) plates and quantitatively measured by enzyme assays. For BACTH assays, we cloned the wild-type or mutant *clpC* genes into the pKT25 or pUT18C vectors, which were then cotransformed into a Δ*cya* E. coli strain (DHT1). As expected, we observed both positive qualitative and quantitative results when assessing homotypic interactions (i.e., wild-type with wild-type) (Fig. 4A and B). Heterotypic interactions between wild-type and mutant ClpC were also analyzed, and these resulted in positive interactions as well (Fig. 4A and B). Importantly, we did not observe interactions between ClpC and ClpX, which served as a negative control for these studies. These results demonstrate the ability of wild-type and mutant ClpC to oligomerize both homotypically and heterotypically. Therefore, we expect that ectopically expressed wild-type and mutant ClpC can potentially form mixed complexes with endogenous ClpC in Chlamydia. This is consistent with previous data for the other chlamydial Clp components and is important for subsequent data interpretation (30).

To confirm oligomerization during infection, we infected HeLa cells with wild-type Ctr L2 and collected protein lysates throughout development and compared samples that had been cross-linked or not using disuccinimidyl suberate (DSS). Then, 25 μg of whole-cell lysates were separated by SDS-PAGE, transferred to polyvinylidene difluoride (PVDF) membranes, and probed with an antibody for endogenous ClpC. Western blot data indicated the presence of a ClpC monomer in the range of the expected size (95 kDa) in both conditions starting at 16 h postinfection (hpi) (Fig. 4C and Fig. S6), consistent with previous data using an alternatively derived antibody (31). Interestingly, in samples treated with DSS, we observed higher order banding consistent with the expected size of both dimers (190 kDa) and hexamers (570 kDa). Additional bands were present in the cross-linked samples, which may reflect potential substrates or other interaction partners. This is under investigation. Immunofluorescence analysis (IFA) of control samples revealed ClpC as early as 10 hpi, a time when transcripts for *clpC* are increasing (Fig. 4D) (31). Overall, these data confirm that ClpC can oligomerize in chlamydial cells to form hexamers necessary for activity.

10.1128/mbio.00075-23.6 FIG S6 Validation of the polyclonal ClpC antibody. Immunoblotting using antibodies of either (A) ClpC, or (B) MOMP for confirmation of Ctr infection. ClpC = 95.2 kDa, MOMP = 42 kDa. Lanes 1 and 3 show whole-cell lysates collected from McCoy cells either uninfected or infected with a wild-type C. trachomatis serovar L2 Bu/434. The negative and positive controls are nonspecific or ClpC specific fractions, respectively, obtained from size exclusion chromatography. Multiple bands in lane 4 are likely a result of ClpC degradation over time. Lane 5 was collected from HeLa cells infected with Ctr:pBOMBL(ClpC) at 20 hpi and subsequently crosslinked with DSS. Download FIG S6, PNG file, 0.2 MB. Copyright © 2023 Pan et al. 2023 Pan et al. https://creativecommons.org/licenses/by/4.0/ This content is distributed under the terms of the Creative Commons Attribution 4.0 International license.

### Overexpression of wild-type ClpC is detrimental to Chlamydia.

**FIG 5:**
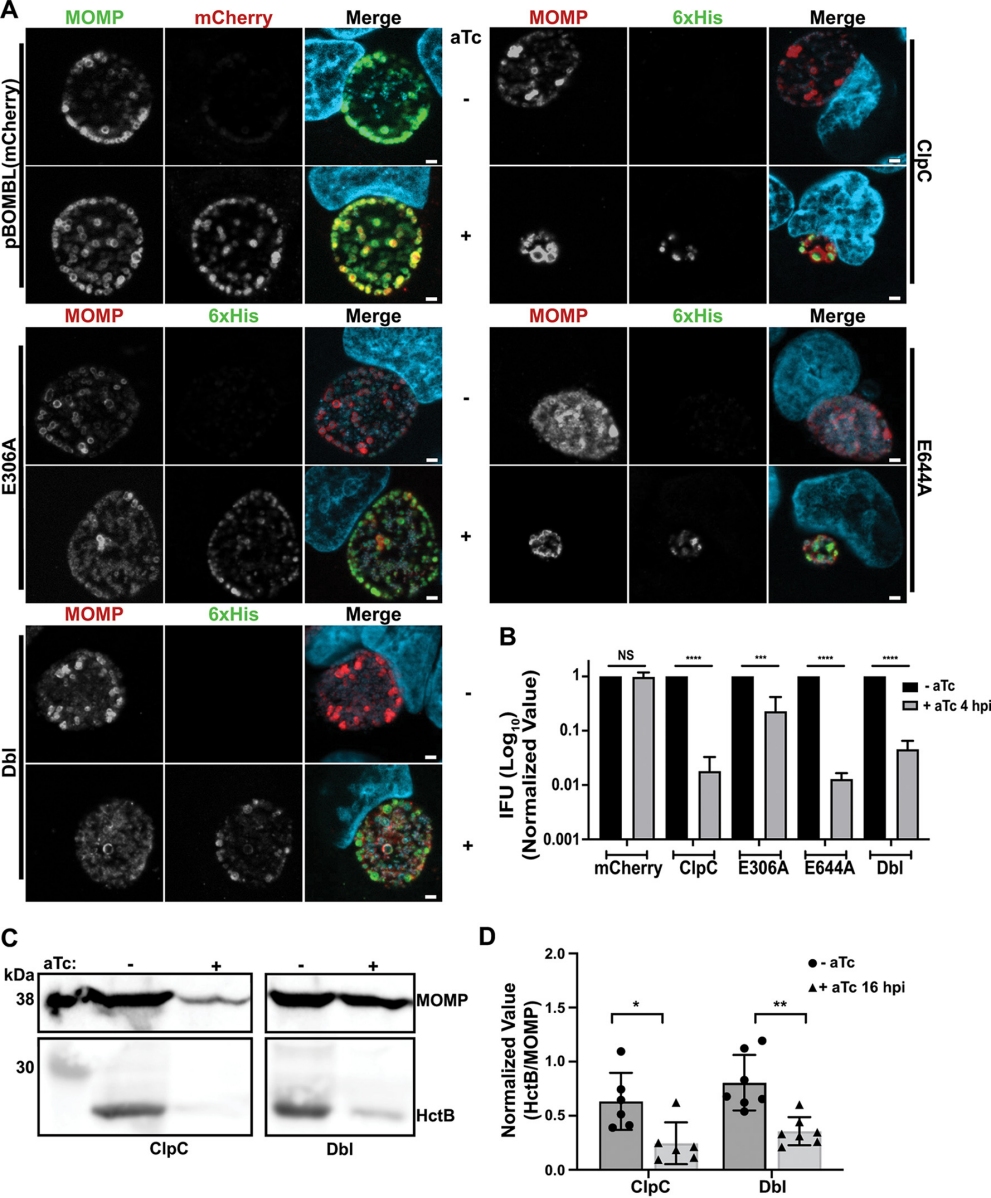
Overexpression of wild-type ClpC disrupts chlamydial growth with a critical function for NBD1 in this activity. (A) Effect of exogenously overexpressed ClpC_6xHis wild-type (ClpC) and mutants (E306A, E644A, Dbl) on chlamydial inclusion size in infected cells analyzed by immunofluorescence. A plasmid expressing mCherry served as negative control. Overexpression was induced or not at 4 hpi with aTc. Samples were fixed at 24 hpi and were stained for MOMP (red for ClpC strains/green for mCherry strain) or 6xHis (green) tagged protein from plasmid induction, and DNA (light blue). Representative images were taken in triplicate on a Zeiss Apotome at ×100 magnification with ×5.5 digital zoom. Scale bar = 2 μm. (B) Quantification of chlamydial growth after expressing or not ClpC, ClpC mutants, or mCherry (vector control). Recovered IFUs of cells infected with each transformant from panel A. Inclusions were identified by counting the GFP-positive inclusions. Data are the average of at least three independent experiments. (C) Effects of ClpC wild type or Dbl mutant overexpression on the levels of HctB (EB-specific protein) and MOMP (loading control for infection) compared to the uninduced controls as determined by immunoblotting. Representative images are shown. Whole-cell lysates were collected at 48 hpi from HeLa cells infected with Ctr exogenously expressing ClpC or Dbl induced at 16 hpi with aTc, and equal amounts of protein from lysates were separated by SDS-PAGE prior to transfer to PVDF for Western blotting. MOMP = 42 kDa, HctB = 23.5 kDa. (D) Densitometric analysis of panel C, where HctB levels were normalized to MOMP levels. Data are the average of at least three independent experiments. The statistical significance analysis was performed via a parametric, unpaired Student’s *t* test (*, *P* < 0.05; **, *P* < 0.01; ***, *P* < 0.001; ****, *P* < 0.0001).

We have previously assessed the effects of overexpression of wild-type and mutant constructs of ClpP1, ClpP2, and ClpX on chlamydial growth and viability (30, 31). In general, overexpression of mutant but not wild-type Clp proteins was detrimental to Chlamydia. To determine the impact of overexpression of ClpC or its mutants, we created plasmids encoding anhydrotetracycline (aTc)-inducible, 6×His-tagged constructs and transformed them into Ctr L2 lacking its endogenous plasmid (-pL2). Similar to endogenous ClpC, we confirmed that the ectopically expressed ClpC or Dbl mutant also formed higher order structures in cell culture infections (Fig. S7). As a control, we also examined the effect of overexpressing mCherry from the same plasmid backbone. HeLa cells were infected with the different strains, ClpC or mCherry expression was induced at 4 hpi (Fig. 5A) or 10 hpi (Fig. S8), and at 24 hpi, cells were subsequently fixed for IFA to determine the effects on bacterial and inclusion morphology or harvested for inclusion forming unit (IFU: a proxy for EBs produced in the initial infection) assays to quantify growth effects (Fig. 5B). For C. trachomatis L2, late gene expression is activated and EBs are produced by 24 hpi (47–49).

**FIG 6:**
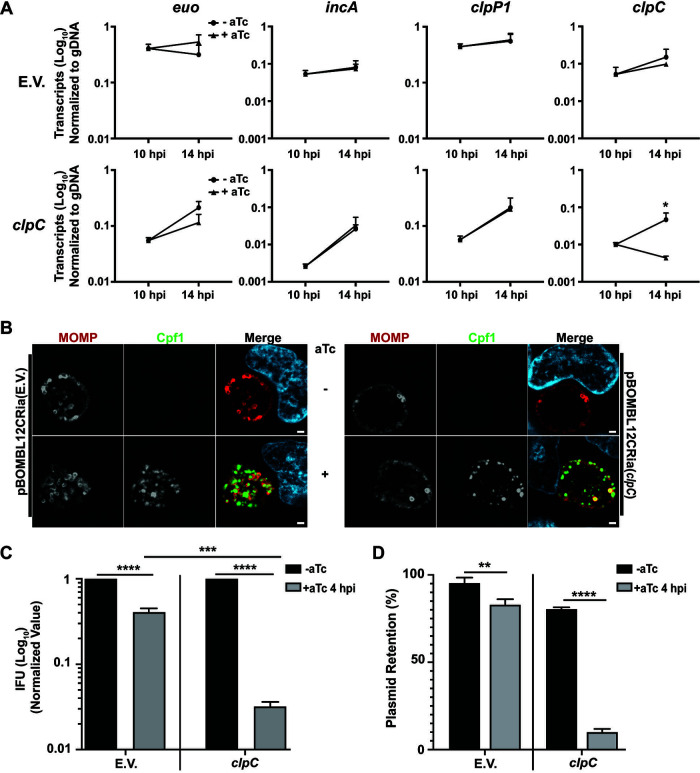
Knockdown of ClpC expression using CRISPR interference is detrimental to C. trachomatis. (A) Levels of *clpC* and control transcripts in strains expressing a *clpC*-targeting CRISPRi or dCas12 alone (i.e., empty vector). Graphs show RT-qPCR data of the indicated transcripts between uninduced and induced samples normalized to genomic DNA (gDNA). (B) Immunofluorescence analyses of the effects of knocking down, or not, *clpC* expression on bacterial morphology and inclusion size. Immunofluorescence of the empty vector (E.V.) or *clpC* knockdown strains where cells were stained for MOMP (red), Cpf1 (for dCas12; green), and DNA (blue) after methanol fixation. Representative images were taken in triplicate on a Zeiss Apotome at ×100 magnification with ×5.5 digital zoom. (C) Quantitative assessment of the effect of *clpC* knockdown (induced at 4 hpi) on chlamydial EB production after 24 hpi. IFU samples were collected in tandem with samples from panel B and quantified on a fresh cell layer as described in the methods section. (D) Plasmid retention during knockdown of *clpC* (induced at 4 hpi) and control condition (E.V.) after 24 hpi. Plasmid retention was examined in aldehyde-fixed samples to preserve GFP signal and reported as the ratio of GFP-positive (marker for the transformation plasmid) to MOMP-positive (total bacteria) inclusions in the IFU assay between uninduced and induced samples from panel C. The statistical significance analysis was performed via a parametric, unpaired Student’s *t* test (*, *P* < 0.05; ***, *P* < 0.001; ****, *P* < 0.0001).

10.1128/mbio.00075-23.7 FIG S7 Expression levels and oligomerization states of ClpC and mutants in cell culture under different conditions. (A) Detection of ClpC_6xHis or mutants in infected cells (induced or not with aTc at 4 hpi, collected at 24 hpi) via an anti-6xHis antibody. Of note, the ClpC image is from a different blot than the ladder and the other three mutants. See also main Fig. 6. (B to E) Exogenously expressed ClpC_6xHis and Dbl mutant can oligomerize in chlamydial cells (induced or not with aTc at 16 hpi, collected at 48 hpi). Western blots of whole-cell lysates for (B) wild-type ClpC_6xHis and (D) Dbl mutant, with IFA controls of overexpressed (C) ClpC_6xHis or (E) Dbl mutant. Some samples were crosslinked as indicated prior to harvesting lysates. Arrows indicate predicted dimer or hexamer bands. A putative degradation product of ClpC within protein samples was consistently observed and is denoted by *. See also main Fig. 4. Expected sizes of ClpC monomer = 95.2 kDa, ClpC dimer = 190.4, ClpC hexamer = 571.2, and MOMP = 42 kDa. C and E, Scale bar = 10 μm. Download FIG S7, PNG file, 1.2 MB. Copyright © 2023 Pan et al. 2023 Pan et al. https://creativecommons.org/licenses/by/4.0/ This content is distributed under the terms of the Creative Commons Attribution 4.0 International license.

10.1128/mbio.00075-23.8 FIG S8 Overexpression of ClpC mutants shows NBD1 is critical for function and essential for chlamydial growth (see also main Fig. 5). (A) Immunofluorescence analysis of the control plasmid expressing mCherry, exogenously overexpressed ClpC or mutants (induced at 10 hpi with aTc). Samples were fixed at 24 hpi and stained for MOMP (in red) as well as for 6xHis (in green) for ClpC and mutants or for mCherry (in green), and for DNA (light blue). Representative images were taken in triplicate on a Zeiss Apotome at ×100 magnification with ×5.5 digital zoom. Scale bar = 2 μm. (B) Recovered IFUs of cells infected with each transformant from (A). Data are the average of at least three independent experiments. The statistical significance analysis was performed via a parametric, unpaired Students T-test (NS = not significant; ****, *P* < 0.0001). Download FIG S8, PNG file, 2.5 MB. Copyright © 2023 Pan et al. 2023 Pan et al. https://creativecommons.org/licenses/by/4.0/ This content is distributed under the terms of the Creative Commons Attribution 4.0 International license.

For IFA, fixed cells were labeled with an anti-6×His antibody to examine induced ClpC expression or directly assessed for mCherry fluorescence (control plasmid), an antimajor outer membrane protein (MOMP) antibody as a marker for individual bacteria, and DAPI to stain DNA. There was no observable impact of overexpressing mCherry on inclusion size or bacterial numbers (Fig. 5A). In contrast to what we observed with the other Clp proteins (30, 31), overexpression of wild-type ClpC resulted in a stark difference compared to the uninduced sample. Whereas typical inclusion sizes were observed in the uninduced conditions, overexpression of wild-type ClpC resulted in noticeably smaller inclusions with fewer organisms. A similar phenotype was observed when overexpressing the E644A mutant (inactivated Walker B motif in NBD2). However, corroborating our *in vitro* results, the inclusion morphology was not obviously altered for the E306A mutant (inactivated Walker B motif in NBD1) or for the Dbl mutant. To assess the generation of viable infectious EBs, we used an IFU assay (equivalent to a CFU assay for most bacteria), which were generally consistent with the IFA data (Fig. 5B). Overexpression of wild-type ClpC and the E644A mutant resulted in a significant (100-fold) decrease in IFU production. Interestingly, the Dbl mutant displayed a significant reduction (~30-fold) in IFUs even though the IFA appeared to be unaffected compared to the uninduced samples whereas overexpression of the E306A mutant resulted in a statistically significant decrease in growth (~5-fold) that was much less than the other mutants. These results indicate (i) that Chlamydia is highly sensitive to increased wild-type or mutant ClpC levels and (ii) that, consistent with our *in vitro* data, the NBD1 is more important for ClpC activity in chlamydial cells.

To further explore why the wild-type and Dbl mutant overexpression strains displayed similar IFU recoveries but disparate morphological effects by IFA, we examined whether secondary differentiation, as assessed by the levels of the EB-associated protein HctB, showed differences in these strains. To this end, we infected cells and induced overexpression at 16 hpi and subsequently collected protein lysates at 48 hpi from both strains and their uninduced controls. Equal amounts of protein from lysates were separated by SDS-PAGE prior to transferring to PVDF for Western blotting. Actin levels were equivalent between lysates (not shown). We induced at 16 hpi to ensure adequate expression of protein in the wild-type strain given the dramatic inhibition in inclusion and bacterial growth and development when overexpressing wild-type ClpC. Given these effects from overexpression, the levels of HctB normalized to MOMP were significantly reduced during ClpC overexpression compared to the uninduced control, as expected (Fig. 5C and D). Similarly, and consistent with the IFU data, the normalized levels of HctB were also reduced in the Dbl mutant when overexpressed (Fig. 5C and D). These data indicate that overexpression of the wild-type ClpC blocks developmental cycle progression (as reflected by smaller inclusions compared to uninduced, low MOMP, and low HctB), whereas overexpression of the Dbl mutant prevents secondary differentiation while having a limited impact on overall inclusion growth and bacterial replication (i.e., inclusion sizes equivalent to uninduced, high MOMP, low HctB).

Since both ClpC and ClpX interact *in vitro* with the same ClpP ortholog in Chlamydia, we wanted to ensure that the effects of ClpC overexpression in cell culture were not a result of titrating ClpP away from ClpX. To test this, we coexpressed both wild-type ClpC with a 6×His tag and wild-type ClpX with a FLAG tag as a transcriptional fusion. We have previously shown that overexpression of ClpX, like ClpP2, has limited effects on bacterial growth or inclusion morphology (30, 31). Thus, any negative effect on inclusion morphology when coexpressing both ClpC and ClpX could be attributed to an effect of ClpC, assuming an equal affinity for the ClpP components. Cells were infected, and expression of the proteins was induced at 4 or 10 hpi before fixation for IFA or collection of samples for IFU assays at 24 hpi. Data from both IFA and IFU assays indicated that there was still a dramatic effect on inclusion morphology and a significant reduction in chlamydial growth (Fig. S9). These results indicate that the negative impact of ClpC overexpression is not likely due to ClpC’s titrating ClpP away from ClpX.

10.1128/mbio.00075-23.9 FIG S9 Overexpression of ClpC is not a result of titrating ClpP away from ClpX. (A) Immunofluorescence analysis of exogenous dual expression of ClpC_6xHis and ClpX_FLAG induced at 4 or 10 hpi with aTc and fixed at 24 hpi. Samples were stained for MOMP (red), 6xHis (green, ClpC) or FLAG (pink, ClpX) tagged proteins from plasmid induction, and DNA (light blue). Representative images were taken in triplicate on a Zeiss Apotome at ×100 magnification with ×5.5 digital zoom. Scale bar = 2 μm. (B) Recovered IFUs of cells infected with each transformant from (A). Data are the average of three independent experiments. The statistical significance analysis was performed via a parametric, unpaired Students T-test (****, *P* < 0.0001). See also main Fig. 5 for comparison. Download FIG S9, PNG file, 0.9 MB. Copyright © 2023 Pan et al. 2023 Pan et al. https://creativecommons.org/licenses/by/4.0/ This content is distributed under the terms of the Creative Commons Attribution 4.0 International license.

### Inhibition of *clpC* expression by CRISPR interference is detrimental to chlamydial growth.

As Ctr was sensitive to increased levels of ClpC, we next wanted to examine the effects of reduced ClpC levels on chlamydial growth using an inducible dCas12 CRISPR interference (CRISPRi) knockdown system developed in our lab (50). The *clpC* gene is transcribed during RB growth and division, and transcript levels peak at 14 to 16 hpi (31). We first validated that knockdown was occurring with a *clpC*-targeting crRNA after dCas12 expression by inducing samples at 10 hpi and collecting nucleic acid samples at 14 hpi (Fig. 6A). RT-qPCR analysis revealed an approximate 75% reduction in *clpC* transcripts, confirming knockdown in the *clpC*-targeting strain, but no significant effects on other control transcripts (i.e., *euo*, *incA*, and *clpP1*). There was no statistically significant effect of dCas12 expression alone (i.e., the empty vector [EV] control without a crRNA) on any of the transcripts we assessed (Fig. 6A). Interestingly, we did not see any changes to inclusion or bacterial morphology by IFA at 24 hpi (after knocking down *clpC* transcription at 4 hpi) compared to the empty vector control expressing dCas12 alone (Fig. 6B). We next examined the effects of *clpC* knockdown by IFU assay at 24 hpi by counting GFP-positive inclusions since GFP is constitutively expressed from the CRISPRi plasmid (Fig. 6C). Under these conditions, we measured a significant, ~30-fold decrease in IFUs in samples where *clpC* transcripts had been knocked down. In addition, there was a significant ~90% reduction in overall plasmid retention after *clpC* knockdown as observed by penicillin susceptible inclusions lacking GFP within our IFU samples (Fig. 6D). The fact that these inclusions were present in the secondary infection for IFU quantification indicates that at least some of the EBs formed during *clpC* knockdown lacked the CRISPRi plasmid. This indicates that Chlamydia is highly sensitive to reductions in ClpC levels and responds by losing both the ability to form functional EBs and the ability to retain the CRISPRi plasmid, rendering subsequent RBs derived from these progeny susceptible to penicillin, which is nonbactericidal in Chlamydia (51, 52). Importantly, overexpression of dCas12 alone had a statistically significant, but biologically limited, 2-fold or less effect on chlamydial growth and plasmid retention (Fig. 6C and D), consistent with a metabolic cost of overexpressing the dCas12 protein, as previously observed (50). Of note, plasmid loss was not observed when overexpressing ClpC (data not shown). Overall, these data, taken together with the overexpression data, indicate that alterations in ClpC levels and/or activity severely disrupt chlamydial growth and development.

## DISCUSSION

Current treatment modalities for chlamydia rely on broad-spectrum antibiotics that can have negative effects on normal microflora within the genital tract (53). Hence, there is an urgent need to develop, in the short-term, better antichlamydial treatment strategies by employing novel targets and mechanisms of antibiotic action and, in the long-term, vaccines capable of preventing infection. Regarding the former, we and others have demonstrated the utility of targeting chlamydial proteases to eradicate infection in cell culture and animal models (31, 32, 54, 55). Interestingly, in terms of new antichlamydial strategies, compounds that were derived from known ClpP-targeting dysregulators were highly effective toward Ctr ClpP and ClpXP activity *in vitro* (32), and one of these compounds was verified to bind the ClpP2 protein in crystallographic data (56). Many compounds, including the one crystallized with ClpP2, prevented chlamydial growth while having a limited impact on other bacterial species (57). Similarly, compounds derived from ClpX-targeting dysregulators were also effective against C. trachomatis (30, 58). Taken together, these data indicate that the Clp system is likely essential for Chlamydia and that targeting the chlamydial Clp protease complex could be a novel strategy for antichlamydial treatment. A more comprehensive understanding of the individual Clp components in Chlamydia will facilitate this goal.

Prokaryotes express a variety of ATP-dependent protease complexes, such as the Clp and Lon proteases, that can perform regulated protein degradation (59–61). For the Clp system, AAA+ unfoldases are critical for recognition and unfolding of substrates into the proteolytic chamber comprised of the barrel-shaped ClpP tetradecamers (5). One such unfoldase is ClpC, a class I unfoldase with two nucleotide binding domains (NBD). ClpC is commonly found in Gram-positive bacteria, where it has been shown to regulate vital cellular processes, including stress responses, genetic competence, cell physiology, differentiation, and even virulence (62). In B. subtilis, ClpC is encoded within an operon containing accessory factors McsA/B that support ClpC substrate targeting by phosphorylating arginine residues on target proteins (60, 63). ClpC recognizes and binds these phosphorylated arginine residues to deliver the substrate to ClpP. That the obligate intracellular Gram-negative pathogen Chlamydia, an organism with a highly reduced genome, would maintain an ortholog to ClpC is intriguing. Based on the characterized functions of ClpC in other bacteria, we hypothesized that ClpC is an important factor for developmental cycle progression and differentiation in Chlamydia. Indeed, ClpC may be essential to Chlamydia, consistent with the failure to obtain a *clpC*::intron mutant (D. Fisher, unpublished observation) and loss of a *clpC*::Tn mutant from a transposon mutant pool during serial passaging (64).

We initiated our studies by performing *in silico* analyses through a bioinformatics approach. Based on sequence alignments, all the key residues and domains associated with substrate recognition and ATPase activity are present in chlamydial ClpC and share high homology with other ClpC orthologs (Fig. 1 and Fig. S1, and S2). Of note, the chlamydial ClpC contains a novel serine-rich linker region between the N-terminal substrate binding domain and the first ATPase domain. Whether this linker can be phosphorylated as a regulatory mechanism or simply provides more flexibility for substrate recognition requires further study. Nonetheless, the observed stringent conservation of domain organization, critical motifs for ATPase activity, and ClpP-binding motif suggests a conserved mechanism of action of both ClpC and ClpX in most bacteria, including Ctr.

Using recombinant proteins of chlamydial ClpC, we confirmed that wild-type ClpC has an *in vitro* dose-dependent intrinsic ATPase activity that was abolished by mutating both glutamic acids in the Walker B NBDs. However, NBD1 contributed most of the intrinsic ATPase activity: the single E306A mutant reduced the activity by ~70% while mutation of the NBD2 (E644A) decreased ATPase activity by only 30%. Given that NBD1 domains are suggested to have a major role in substrate unfolding, this result is perhaps not surprising, but indeed, it contrasts with previous findings on B. subtilis ClpC where Walker B NBD2 contributed to the majority (85%) of the intrinsic ATPase activity (4, 65). Chlamydial ClpC also possessed some chaperone-like activity as it reduced aggregation of heat-treated luciferase. Again, the NBD1 was more important for this activity. Interestingly, we observed that the activity of chlamydial ClpC was roughly half that of the B. subtilis ortholog, and, unlike B. subtilis ClpC, its chaperone activity could not be enhanced by the addition of bsMcsAB, which was used as a surrogate for the chlamydial McsAB due to ongoing difficulties purifying the proteins. Therefore, our data suggest differences for the individual activity and function of ClpC in Chlamydia and B. subtilis, which may relate to the different roles of Walker B NBD1 and NBD2 in both enzymes and in the unique biology of Chlamydia.

Similar to our previous observation for chlamydial ClpX (32), ClpC also formed an active protease complex with a mixed ClpP1P2 tetradecamer *in vitro*. No functional proteolytic activity was observed when ClpC was present with either ClpP1 or ClpP2, individually. Our data revealed that interaction of ClpC with the ClpP1P2 core is mediated via the ClpP2 protein, thereby identifying ClpP2 as the central docking partner for both AAA+ ATPases, ClpC and ClpX, in Chlamydia. In this asymmetric complex, ClpP1 contributes to enzymatic activity together with ClpP2 (32) and may further serve as a crucial structural element for the assembly of the proteolytically competent ClpP core, like mycobacterial Clp (66–69), which may provide an explanation for the apparent functional interdependence of chlamydial ClpP1 and ClpP2. This is further supported by cluster analyses which locate ClpP1 far outside the ClpP “super cluster” (Fig. 1C), indicating pronounced differences of ClpP1 to ClpP2 and other ClpP orthologs that may explain the lack of binding to ClpC and ClpX.

Interestingly, our *in vitro* protease assays also revealed that the standard β-casein substrate typically used in such assays was not degraded by ClpCP2P1. Rather, an arginine-phosphorylated form of β-casein was necessary for degradation to be detected. In an *in vitro* assay, ClpCP2P1 did not degrade SsrA-tagged GFP (P. Sass, unpublished observation), a ClpX substrate (29, 30, 32), suggesting different substrate pools for ClpC and ClpX in Chlamydia. As noted above, ClpC is typically encoded with McsAB in an operon (62, 70). Intriguingly, Chlamydia also possesses McsAB orthologs that are encoded as an operon but separated from *clpC* in the chromosome. The Ctr *mcsAB* operon is flanked by tRNAs, suggesting that it may have been horizontally acquired. Further work is necessary to determine the function of these proteins and whether arginine phosphorylation is occurring during the chlamydial developmental cycle. One intriguing possibility under investigation in our lab is that phosphorylation of arginine residues on critical substrates leads to their degradation to trigger differentiation. Such a model is consistent with the described activity of the ClpC/McsAB system in other bacteria (63, 70). However, to evaluate this in Chlamydia, we need to identify potential substrates recognized by the ClpC unfoldase, which is complicated by its obligate intracellular nature.

We next assessed the function of ClpC during chlamydial growth and development within cell culture experiments. We first examined the oligomerization state of endogenous ClpC during the developmental cycle using cross-linkers to preserve higher order complexes since ClpC functions as a hexamer in other bacteria. With this approach, we observed that both dimers and larger complexes, consistent with a hexamer, were detected starting at 16 hpi, the earliest time at which we detected endogenous ClpC. Notably, the bulk of protein was in a monomeric form, suggesting that ClpC may require activation to form a complex in the chlamydial cell. This would be consistent with other systems (7, 63). We also detected additional bands in the cross-linked samples that may represent substrates bound to ClpC, but this requires further investigation and is a major goal moving forward.

To evaluate the effects of altering ClpC levels in Chlamydia, we created transformants carrying plasmids with inducible constructs encoding wild-type or NBD1/2 mutations (separately and together). Based on our prior studies of chlamydial ClpX and ClpP, we had anticipated that overexpressing the wild-type ClpC would have a negligible impact on chlamydial growth whereas the mutant ClpC would impair developmental cycle progression (30, 31). Surprisingly, we observed the opposite: a drastic reduction in chlamydial growth when overexpressing the wild-type ClpC but less of an effect when overexpressing the Dbl (E306A/E644A) Walker B mutant. Interestingly, overexpression of the Dbl ClpC mutant resulted in normal morphology but reduced IFU yields, suggesting that production of viable EBs may be impaired as we noted for the ClpX Walker B mutant (30). Further, data from our cell culture experiments confirmed the importance of the NBD1, as overexpressing the E306A mutant had only a slight negative impact on chlamydial growth whereas overexpressing the E644A mutant resulted in a similar phenotype as the wild-type ClpC overexpression. Given that both ClpX and ClpC interact with ClpP2 *in vitro* to form an active protease, one interpretation of these data are that higher levels of ClpC may outcompete ClpX for ClpP2, resulting in ClpX no longer accomplishing its function. Nonetheless, coexpressing both unfoldases resulted in the same phenotype as expressing ClpC alone, suggesting that competition between unfoldases for ClpP binding does not explain the ClpC overexpression phenotype. Rather, ClpC binding of an essential substrate(s) may lead to its degradation or sequestration. However, we cannot exclude that the unfoldases may have differential binding affinities for the ClpP complex, which requires further investigation.

As a complementary approach to our overexpression studies, we also implemented a CRISPR interference strategy developed in our lab for the inducible repression of *clpC* transcription (50). We confirmed successful knockdown of *clpC* transcript levels at the peak phase of its expression during the developmental cycle. Our results indicate that Chlamydia is highly sensitive to reduced ClpC levels as we observed a significant loss of both IFUs and the plasmid encoding the CRISPRi system in EBs that were produced, which resulted in the susceptibility of the organism to the selection agent (penicillin) evident during the titration step of the IFU assay. Not surprisingly given this observation, we determined that reduced ClpC levels resulted in decreased chlamydial progeny production. These data were similar to the effects of overexpression of the Dbl mutant, which is also expected to disrupt ClpC-mediated degradation of its substrates. Collectively, these data suggest that the inability of ClpC (by its absence or impairment) to target specific substrate(s) for degradation by ClpP or for possible chaperone functions impairs growth and/or differentiation in Chlamydia. Overall, both the overexpression and knockdown data suggest that the activity of ClpC must be tightly regulated to ensure proper developmental cycle progression.

In sum, the current work has helped to significantly advance our understanding of the Clp system in an important obligate intracellular pathogen. This study has demonstrated (i) that chlamydial ClpC has the function and activity of a canonical ClpC with the ability to recognize a phosphoarginine-tagged substrate for subsequent degradation by ClpCP2P1, and (ii) that alterations to ClpC levels have dramatic negative impacts on chlamydial growth and viability. Taken together, this indicates ClpC is likely essential to Chlamydia and represents a potential target for the development of antichlamydial compounds.

## MATERIALS AND METHODS

### Strains and cell culture.

Recombinant Clp proteins from C. trachomatis were expressed using E. coli BL21(DE3) *dAPX-1*, as previously described, to prevent undesired copurification of E. coli Clp proteins (32). Recombinant McsA and McsB of B. subtilis (bsMcsAB) and McsB of Geobacillus stearothermophilus (gsMcsB) were expressed using E. coli strain *C43*. Unless stated otherwise, cells were cultivated in lysogeny broth (LB) supplemented with ampicillin (100 μg/mL) at 37°C under vigorous agitation. The human epithelial HeLa cells were used for all studies involving cell culture (overexpression, gDNA, protein extractions, plaque purification, transformation). HeLa cells were routinely passaged in Dulbecco’s modified Eagle’s medium (DMEM; Gibco/Thermo Fisher) with 10% fetal bovine serum (FBS; Sigma, St. Louis, MO) and were verified to be mycoplasma free by a LookOut mycoplasma PCR detection kit (Sigma). C. trachomatis serovar L2 EBs (25667R) (kind gift of Ian Clarke, University of Southampton) that lacked endogenous pL2 plasmid were prepared and used for transformation (71).

### Bioinformatic analysis.

Gene sequences of Chlamydia trachomatis (Bu 434 and A/HAR-13), Chlamydia psittaci (6BC genotype A), Chlamydia pneumoniae (AR39), Chlamydia suis, and Parachlamydia acanthamoebae were obtained from STDGEN database (http://stdgen.northwestern.edu) or KEGG genome browser (72–74). The RefSeq protein sequences for Chlamydia trachomatis (D/UW-3/CX), Chlamydia muridarum, Staphylococcus aureus (PS47), Mycobacterium tuberculosis (H37Rv), and B. subtilis (strain 168) were acquired from the NCBI protein database (http://www.ncbi.nlm.nih.gov/proteins/). Pairwise protein alignments for ClpC to find sequence identity were performed using the NCBI Protein BLAST function (https://blast.ncbi.nlm.nih.gov/Blast.cgi) (75). Multiple sequence alignments were performed using Clustal Omega (76) with default settings and formatted for presentation using Jalview version 2.11.1.4 (77). Homology relationship plots based on Cluster Analysis of Sequences (CLANS) (39) were computed using ClpX, ClpC, and ClpP amino acid sequences and the BLOSUM62 scoring matrix. Input included 597 unique ClpP and 493 unique ClpX sequences extracted from the UniProtKB/Swiss-Prot database (reviewed sequences). Input ClpC sequences consist of 34 unique sequences from the UniProtKB/Swiss-Prot database (reviewed sequences) and 1,637 unique sequences (800 to 950 aa in length) from the UniProtKB/TrEMBL (unreviewed) database. BLAST HSP E values up to 1e-10 were extracted for calculating pairwise attraction values. For iterative cluster formation, the *P* value threshold was set to 1e-75. To examine predicted 3D structure, PBD files were acquired from the Phyre2 website (http://www.sbg.bio.ic.ac.uk/phyre2/html/page.cgi?id=index) (78). The acquired PBD file was then 3D-modeled using SWISS-MODEL, available on the ExPASy server (79–81).

### Plasmid construction.

A full list of primers used in this study is provided in Table S1 within the supplemental material. Cloning of the ClpC expression plasmid was achieved by ligating PCR-amplified C. trachomatis *clpC* (CT_286, C. trachomatis D/UW-3/CX, [GenBank accession no. NC_000117.1]; shows 2 nonsynonymous changes not associated with critical functions in ClpC compared to serovar L2) into the pET-11a vector, which allows for the expression of fusion proteins with a C-terminal Strep tag, using the NEBuilder system (New England Biolabs; NEB). Suitable primers for PCR were calculated by the NEBuilder assembly tool 2.0 and are listed in Table S1. For the construction of expression plasmids encoding bsMcsA (ORF BSU_00840) and bsMcsB (ORF BSU_00850) of B. subtilis *168* (GenBank accession no. NC_000964.3), PCR-amplified inserts were obtained by using the primers listed in Table S1. The *mcsB* gene of *G. stearothermophilus* (*gsmcsB*) was amplified from pET-21a-gsmcsB_his (82) for subcloning using the primers listed in Table S1. All resulting PCR fragments were cloned into a modified pET-11a vector featuring two *Bsa*I nonidentical restriction sites as a universal, nonpalindromic polylinker, followed by a C-terminal Strep(II) tag sequence (pET-MP3) (32).

10.1128/mbio.00075-23.10 TABLE S1 List of plasmids, strains, and primers. Download Table S1, PDF file, 0.2 MB. Copyright © 2023 Pan et al. 2023 Pan et al. https://creativecommons.org/licenses/by/4.0/ This content is distributed under the terms of the Creative Commons Attribution 4.0 International license.

Site-directed mutagenesis was achieved by using the QuikChange II site-directed mutagenesis kit (Agilent) according to the manufacturers protocol. Previously constructed plasmids encoding the hydrophobic pocket mutant proteins ClpP1_L186T_ and ClpP2_I190T_ (32) were used as templates to introduce additional mutations V57A and M77A for ClpP1_L186T_ and F63A and F83A for ClpP2_I190T_. See Table S1 for primers used for site-directed mutagenesis. The resulting ClpP1 and ClpP2 hydrophobic pocket triple mutants were referred to as ClpP1_H3_ and ClpP2_H3_. In the same manner, construction of ClpC Walker B motif mutants (E306A, E644A) was performed using the primers listed in Table S1. The resulting ClpC Walker B motif single mutants were referred to as ClpC_E306A_ and ClpC_E644A_, whereas the ClpC Walker B motif double mutant was referred to as ClpC_Dbl_. Sequence identity of all resulting constructs was verified by Sanger sequencing (LGC genomics).

BACTH constructs made for homo- and heterotypic interaction analysis were created using the HiFi Cloning (NEB) protocol. NEBuilder assembly tool was used to generate primers (http://nebuilder.neb.com). One set of primers each were designed for PCR amplification to insert products into either pKT25 or pUT18C (46). C. trachomatis L2 (Bu 434) genomic DNA was used as the template for wild-type ClpC, and the pST25 Q5 mutagenesis plasmid (shown below) as the template for ClpC mutant PCR amplification with primers. PCR amplified products were confirmed for length by agarose gel electrophoresis. BACTH plasmids were generated by taking PCR amplified product and inserting them into the backbone of pKT25 or pUT18C cut with BamHI and KpnI. These HiFi reactions were transformed into DH5αI^q^ E. coli (NEB), and isolated plasmid was verified by restriction enzyme digest and sequencing by Eurofins Genomics. The sequence verified plasmids were then used for interaction studies in BACTH (see below).

Constructs made for chlamydial transformation were created using the HiFi Cloning protocol. NEBuilder assembly tool was used to generate primers. Primers were designed to add a poly histidine (6×His) or FLAG tag to the gene of interest being inserted on the 3′ end of the overlap into the shuttle vector. C. trachomatis L2 (Bu 434) genomic DNA was used as the template for PCR amplification with primers, and products were confirmed for length by agarose gel electrophoresis. Overexpression plasmids were generated by first taking PCR amplified products and inserting them into the backbone pBOMBL::L2 [50] cut with EagI and KpnI to remove the mCherry gene. pBOMBL::L2 is a derivative of pBOMB4-Tet::L2 (kind gift of T. Hackstadt, NIH) with a modified weakened ribosomal binding site to reduce leaky expression and off target effects when not induced. The pBOMBL plasmid encodes a constitutively expressed GFP that can be used to monitor transformed bacteria. The HiFi reactions were transformed into DH10β E. coli, and isolated plasmid was verified by restriction enzyme digest and sequencing by Eurofins Genomics. The sequence verified plasmids were transformed into C. trachomatis (see below).

To generate mutations of the ClpC Walker B motifs, Q5 mutagenesis (New England BioLabs) was used. Primers were designed encoding either the E306A or E644A mutation of the Walker B motifs for PCR linearization of the plasmids. The ClpC BACTH construct pST25-ClpC was used as the template for both the PCR amplifications, which were recircularized by a kinase-ligase-DpnI (KLD) reaction. These reactions were then transformed into DH5α E. coli for plasmid generation. The plasmids were isolated, and mutations and sequence were confirmed by Sanger sequencing (Eurofins Genomics) prior to use in the BACTH system. These plasmids served as the templates for PCR products to move mutant *clpC* alleles into pBOMBL::L2, pKT25, or pUT18C.

### Protein expression.

Expression and purification of recombinant chlamydial ClpP1 and ClpP2 was carried out as previously described (32). E. coli cultures containing the expression plasmids for either ctClpC, bsMcsA, bsMcsB or gsMcsB were grown until midlog exponential phase (optical density at 600 nm [OD_600_.] of 0.6 to 0.7). Upon induction of expression by adding 1 mM isopropyl-β-d-thiogalactopyranoside (IPTG), E. coli cultures were further shaken at 18°C overnight. The following purification steps were executed at 4°C. After centrifugation, cell pellet was resuspended in prechilled buffer A (20 mM Tris/HCl, pH 8) supplemented with cOmplete protease inhibitor (Roche) and 5 mM EDTA. Cell disruption was performed by mechanical impact force using accelerated glass beads (150 to 212 μm, Sigma) in a PreCellys homogenizer (Precellys Evolution, Bertin Technologies). Cell debris was pelleted via centrifugation. Remaining supernatant was further filtered using 0.45- and 0.2-μm membrane filters (Sarstedt). Filtered lysates with Strep-tagged proteins were applied onto StrepTrap HP 1-mL columns (GE Healthcare). Subsequent protein separation was conducted using the ÄKTA start chromatography system (GE) according to the respective manufacturer’s protocols with following modifications: To prevent protein precipitation, 5 mM DTT were added to both the washing buffer (100 mM Tris/HCl, 150 mM NaCl, pH 8.0) as well as the elution buffer (100 mM Tris/HCl, 150 mM NaCl, 2.5 mM desthiobiotin, pH 8.0) for ctClpC purification. For bsMcsA, bsMcsB and gsMcsB, 10 mM DTT were added to the washing and the elution buffers. Buffer exchange was performed via centrifugal filters (Amicon Ultracel-30K, Merck) using buffer A supplemented with 30% glycerol and 5 mM DTT. Concentration and purity of desired proteins were determined via SDS-PAGE analyses followed by Bradford assays using a bovine serum albumin (BSA) standard curve.

### Arginine phosphorylation of β-casein.

To obtain the ClpCP protease substrate β-casein-pArg, enzymatic arginine phosphorylation of β-casein was performed in buffer BS (50 mM Tris/HCl [pH 8], 25 mM MgCl_2_, and 100 mM KCl, 2 mM DTT) in a total volume of 500 μL that was supplemented with an ATP regeneration system (2 mM ATP, 5 mM creatine phosphate, 2 U creatine phosphokinase). Then, 5 μM arginine kinase (bsMcsAB or gsMcsB) and 50 μM β-casein were added accordingly. The phosphorylation reaction mixture was incubated for 5 h at 30°C. Reverse purification using Strep-tactin resin was employed to bind and remove bsMcsAB or gsMcsB from the flowthrough. Anti-pArg antibodies were used to verify arginine phosphorylation of β-casein via dot blotting (Fig. S4).

### *In vitro* casein degradation assays.

Degradation of the protease substrate β-casein-pArg was performed in buffer PZ (25 mM HEPES, 200 mM KCl, 5 mM MgCl_2_, 1 mM DTT, 10% [vol/vol] glycerol, pH 7.6). Each reaction sample contained 2 μM bsMcsA, 2 μM bsMcsB, and 5 μM β-casein-pArg. Next, 6 μM total ctClpP in an equimolar ratio (total amount ctClpP1 and/or ctClpP2) and 2 μM ctClpC were added accordingly. Samples of 10 μL were taken from each reaction at specified time points during incubation at 32°C. Substrate degradation was halted by adding sample loading buffer containing LDS (4X Bolt LDS Sample Buffer, Invitrogen) and heating at 80°C. Degradation of the β-casein-pArg substrate was then analyzed via SDS-PAGE and subsequent Coomassie staining.

### *In vitro* ClpC basal ATPase activity assay.

Quantitative determination of the basal ATPase activity of ClpC was performed using the ADP-Glo system (Promega) in 384-well plates, which measures ADP formed from an ATPase reaction. In this assay, unreacted ATP is removed after the ATPase reaction was stopped. Then, the remaining ADP is converted into ATP, which is used to generate luminescence in a luciferase reaction. The luminescence generated correlates with ATPase activity. ATPase reactions were carried out with 50 nM ultrapure ATP plus various concentrations of ctClpC in buffer PZ. ATPase activity was determined according to the kit manual.

### *In vitro* ClpC chaperone activity assay.

Quantitative analysis of ctClpC-mediated protection of luciferase aggregation during heat shock was adapted from Andersson et al. (83). Luciferase from Photinus pyralis (0.2 μM) was heat-inactivated at 43°C for 15 min in the presence of ctClpC (0.4 μM) in buffer PZ or of bsClpC (0.4 μM) in buffer BS, both supplemented with 2 mM ATP. Adapter proteins (bsMcsA, bsMcsB and bsMecA) were added in an equimolar manner as indicated. β-casein (0.4 μM) as a substitute for ClpC served as negative control. After heat inactivation, all samples were immediately moved onto ice. Then, 1 μL from each reaction sample was transferred into 199 μL buffer PZ (for ClpC) or buffer BS (for bsClpC) containing 400 μM d-luciferin and 20 mM ATP. Absolute luminescence was measured at the *t* = 5 s peak intensity.

### Determining protein-protein interactions with the BACTH system.

To test protein-protein interaction between wild-type ClpC and mutants, we utilized the bacterial adenylate cyclase-based two-hybrid (BACTH) assay (46). Genes to be examined were fused to one of either catalytic subunit, denoted as T18 and T25, of the B. pertussis adenylate cyclase. When the catalytic subunits are in close proximity, they can then reconstitute adenylate cyclase activity and allow growth of Δcya DHT1 E. coli (84) on minimal medium with maltose. Both wild-type and mutant *clpC* genes were cloned into pKT25 or pUT18C vectors for testing of both homotypic and heterotypic interactions (see plasmid construction above). Each of the pKT25 and pUT18C plasmids were cotransformed into chemically competent DHT1 E. coli cells, and the cells were plated on a double antibiotic minimal M63 medium selection plate, which was supplemented with 0.5 mM IPTG for induction of the proteins, 40 μg/mL 5-bromo-4-chloro-3-indolyl-β-d-galactopyranoside (X-Gal), 0.04% casein hydrolysate, and 0.2% maltose. For a positive control, leucine zipper motifs were used in both pKT25 and pUT18C backgrounds with the appropriate antibiotic selection plates, as they have previously been shown to interact (85). Blue colonies indicating positive interactions were screened using the β-galactosidase assay. Positive colonies were chosen randomly and grown in M63 minimal medium with the appropriate antibiotics: 0.1% SDS and chloroform were then used to permeabilize the bacteria prior to addition of 0.1% o-nitrophenol-β-galactoside (ONPG), and 1 M NaHCO_3_ was used to halt the reaction after precisely 20 min of incubation time at room temperature. An absorbance at 405 nm wavelength was recorded and normalized to bacterial growth (OD_600_), the dilution factor, and the time (in minutes) of incubation prior to halting the reaction. The totals were reported in Miller units of β-galactosidase activity.

### Purification of recombinant ClpC for antibody generation.

The *clpC* gene from C. trachomatis L2 was cloned into pLATE31 (Invitrogen) to generate a C-terminal 6×His-tagged construct. The vector was sequence verified and transformed into E. coli BL21(DE3) *dAPX-1* for protein purification. Purification was performed using 2 L cultures based on the protocol described in reference (37). Samples were induced with 100 mM IPTG and incubated with shaking for 20 h at 18°C. Cultures were pelleted and frozen at −80°C prior to purification. Samples were suspended in buffer A (25 mM Tris base [pH 7.5], 300 mM NaCl, and 20 mM imidazole), sonicated, ran through a 0.45-μm filter, and finally ran on an ÄKTA start chromatography system (GE). Sample was applied to a HisTrap FF 1 mL column. Proteins were eluted from the resin using buffer B (25 mM Tris base [pH 7.5], 300 mM NaCl, and 300 mM imidazole). Protein was concentrated and smaller proteins were removed using a Millipore Amicon Ultra 15 filtration unit (30 kDa cutoff). Protein was analyzed by SDS-PAGE to assess quality, then was purified further using a size exclusion chromatography (SEC) column on the ÄKTA. Purified protein from SEC was separated on SDS-PAGE to isolate appropriate fractions and was once again concentrated using a Millipore Amicon Ultra 15 filtration unit (30 kDa cutoff). Samples were sent to Thermo Fisher for polyclonal antibody generation in rabbits. Antibody specificity was determined by IFA and Western blot comparing specific, and nonspecific SEC fractions, and uninfected or infected McCoy Cells (see Fig. S6).

### Chlamydial transformation.

The following protocol was a modification to the method developed by Mueller et al. (86). For transformation, 10^6^ C. trachomatis serovar L2 EBs (25667R), naturally lacking the endogenous L2 plasmid, were incubated with 2 μg of plasmid in a volume of 50 μL CaCl_2_ at room temperature for 30 min. Each reaction was sufficient for a confluent monolayer of HeLa cells for a single well of a six-well plate, plated 1 day prior at 1 × 10^6^ cells. The 50 μL of transformant was added to a 1-mL overlay of room temperature Hanks’ balanced salt solution (HBSS) per well, followed by addition of 1 mL of HBSS per well. The six-well plate was centrifuged at 400 × *g* for 15 min at room temperature, and the beginning of this step was marked as the time of infection (0 hpi). Following centrifugation, the plate was incubated for 15 min at 37°C. At the end of the incubation period, the inoculum was aspirated and replaced with antibiotic-free DMEM containing 10 μg/mL gentamicin and 10% FBS. At 8 hpi, the medium was removed and replaced with DMEM containing 1 μg/mL cycloheximide, 10 μg/mL gentamicin, 1 U/mL penicillin, and 10% FBS. Cells infected with transformants were passaged every 48 h by scraping cells from the plate and collecting the contents in a 2 mL microcentrifuge (mcf) tube. The mcf was then centrifuged at 17,000 × *g*, and the supernatant was aspirated, and the pellet resuspended in 1 mL HBSS. The resuspended pellet was then centrifuged again at 400 × *g* to remove host cell debris, and the inoculum was added dropwise to 1 mL of HBSS on a new monolayer of HeLa cells. If a population of penicillin-resistant bacteria was established, then EBs were harvested by centrifuging scraped cells at 17,000 × *g*, aspirating supernatant, and resuspending in sucrose-phosphate (2SP) (71) and spun down at 400 × *g*. The EBs in the supernatant were then collected, and frozen at −80°C prior to stock titration. Plasmids were isolated from the strains using the Dneasy kit (Qiagen), retransformed into E. coli, and reisolated to verify insert by digest and sequencing.

### Western blot detection of endogenous HctB and ClpC or overexpressed ClpC_6×His.

Crosslinking was performed for infections with a multiplicity of infection (MOI) of 1 with either wild-type Ctr L2, pBOMBL-ClpC_6×His::L2, or pBOMBL-ClpC_Dbl_6×His::L2 in a 6-well culturing plate. The pBOMBL-ClpC_6×His::L2 and pBOMBL-ClpC_Dbl_6×His::L2 transformants were induced at 16 hpi to reduce the drastic effects that were seen on inclusion morphology and bacteria number when induced at earlier time points. At time of sample collection, wells were washed three times with HBSS, and a separate well for IFA was fixed with MeOH as a control to monitor infection. Medium was aspirated and replaced with 1 mL HBSS pH 7.6 to better facilitate cross-linking. Disuccinimidyl suberate (DSS) in DMSO (100 mM) was added to cross-linked samples for a final concentration of 0.25 mM DSS. DMSO was added at the same volume to serve as vehicle control for noncrosslinked samples. Six-well plates were placed at 4°C on ice for at least 1 h, aspirated, and 0.5 M Tris pH 7.6 was added to a final concentration of 20 mM per well for 10 min to quench the reaction. Samples were harvested in denaturing cell lysis buffer, quantified for protein using an EZQ protein quantification kit (Invitrogen), and equal amounts of protein were separated on an 8% SDS-PAGE gel with either a Hi-Mark prestained protein standard ladder (Thermo Fisher) or Chameleon Duo prestained protein ladder for kDa sizes. Protein was transferred to PVDF membrane for Western blot and stained with rabbit anti-ClpC antibody (for wild-type Ctr L2), rabbit anti-6×His (for pBOMBL-ClpC_6×His::L2 and pBOMBL-ClpC_Dbl_6×His::L2 transformants; Abcam), or rabbit anti-HctB (for wild-type Ctr L2; kind gift of T. Hackstadt) and goat anti-MOMP of Chlamydia (Meridian Bioscience). Donkey secondary antibodies conjugated to fluorophores were used to detect rabbit (680 nm) and goat (800 nm) primary antibodies (Licor). Labeling was observed on an Azure Biosystems c600 instrument using the NIR autoexposure setting. The Hi-Mark ladder does not have an 800-nm readout for the ladder, so blots were superimposed to position the ladder correctly in the 800-nm channel from the 680-nm channel when used.

### Determining the effect of overexpression of wild-type and mutant ClpC proteins or knockdown of *clpC* via immunofluorescence and inclusion-forming unit analysis.

C. trachomatis transformed with either the pBOMBL-ClpC_6×His::L2 or mutants, the dual expression plasmid pBOMBL-ClpC_6×His-ClpX_FLAG, or pBOMBL12CRia(*clpC*) under the control of an aTc-inducible promoter were used to infect a monolayer of HeLa cells on coverslips utilizing penicillin as a selection agent. Samples were induced with increasing concentrations of aTc at either 4 or 10 hpi and were fixed with methanol after a 20 h or 14 h pulse (24 hpi). Fixed cells were incubated with goat anti-MOMP primary antibody (all IFA samples), mouse anti-6×His (overexpression plasmids), rabbit anti-FLAG (pBOMBL-ClpC_6×His-ClpX_FLAG only; Sigma-Millipore), or mouse anti-Cpf1 (pBOMBL12CRia[clpC]; Sigma-Millipore). Donkey antigoat Alexa Fluor 594-conjugated secondary antibody were used for visualization of the organisms for experiments. A donkey antimouse Alexa Fluor 488-conjugated secondary antibody was used for visualization of ClpC and mutants as well as dCas12, respectively, for expression and localization. To visualize ClpX_FLAG, a donkey antirabbit Alexa Fluor 647-conjugated secondary antibody was used to visualize expression and localization. Lastly, samples were stained with 4’,6-diamidino-2-phenylindole (DAPI) to visualize the host and bacterial cell DNA. Representative images were taken on a Zeiss LSM 800 confocal laser scanning microscope with a 100× objective, then 5.5× digitally zoomed and adjusted for color and brightness with Zen software (blue edition, version 3.3).

To assess the effects of wild-type and ClpC mutant overexpression, the inclusion forming unit (IFU) assay was used. HeLa cells were infected in triplicate, as described above for IFA samples. IFUs were harvested by scraping sample wells in 2SP, pooling triplicate sample wells together, and freezing at least overnight at −80°C. Samples were then removed, vortexed, and serially diluted in HBSS and titrated in duplicate directly onto a new monolayer of HeLa cells. Following 24 to 40 h of incubation, the samples were fixed and stained with goat anti-MOMP primary antibody, and a donkey antigoat Alexa Fluor 594-conjugated secondary antibody for visualization of the bacteria within inclusions. An aldehyde fix was used to retain GFP fluorescence if plasmid retention was to be observed and followed by MeOH permeabilization. Ten fields of view were counted for each duplicate well at ×20 magnification, giving a total of 20 fields of view per experiment. Three independent experiments were performed, and the totals for each experiment were averaged. Displayed values were expressed as a percentage of the uninduced sample to provide an internal control. For statistics, a parametric, unpaired Student’s two-tailed *t* test was used to compare the induced samples to the uninduced control and was performed using the averages of each biological replicate.

### Knockdown of endogenous C. trachomatis ClpC.

Two wells of a six well plate per condition were infected using C. trachomatis transformed with pBOMBL12CRia(*clpC*)::L2 at an MOI of 1.0. At 10 hpi samples were induced or not with 2 nM aTc. For each designated time point, RNA was collected using TRIzol reagent (Invitrogen) and extracted using chloroform with the aqueous phase precipitated with an equal part of isopropanol, according to the manufacturer’s instructions (Thermo Fisher). DNA was removed from RNA samples through rigorous DNA-free treatment (Thermo Fisher) before 1 μg was reverse transcribed with Superscript III reverse transcriptase (RT; Thermo Fisher). Equal volumes of cDNA were used for qPCR. For genomic DNA, cells were trypsinized and collected by centrifugation for 5 min at 400 × *g* and resuspended in phosphate-buffered saline (PBS) then stored at −20°C until further processing. Frozen samples were thawed and subjected to a DNeasy blood and tissue kit (Qiagen) to isolate C. trachomatis DNA according to the manufacturer’s instructions; 5 ng/μL was used for qPCR. Transcripts and genomic DNA were quantified by qPCR in 25-μL reaction mixtures using 2X SYBR green master mix in an ABI700 thermal cycler in comparison to a standard curve generated from purified C. trachomatis L2 genomic DNA. Transcripts were then normalized to genomic DNA for results and the induced samples normalized to the uninduced samples for the same time points.
