# Supplementary figures and images for: Molecular Characterization of the ClpC AAA+ ATPase in the Biology of Chlamydia trachomatis
Source: mBio. 2023 Mar 28;14(2):e00075-23. doi: 10.1128/mbio.00075-23 (PMC10128030; doi:10.1128/mbio.00075-23)

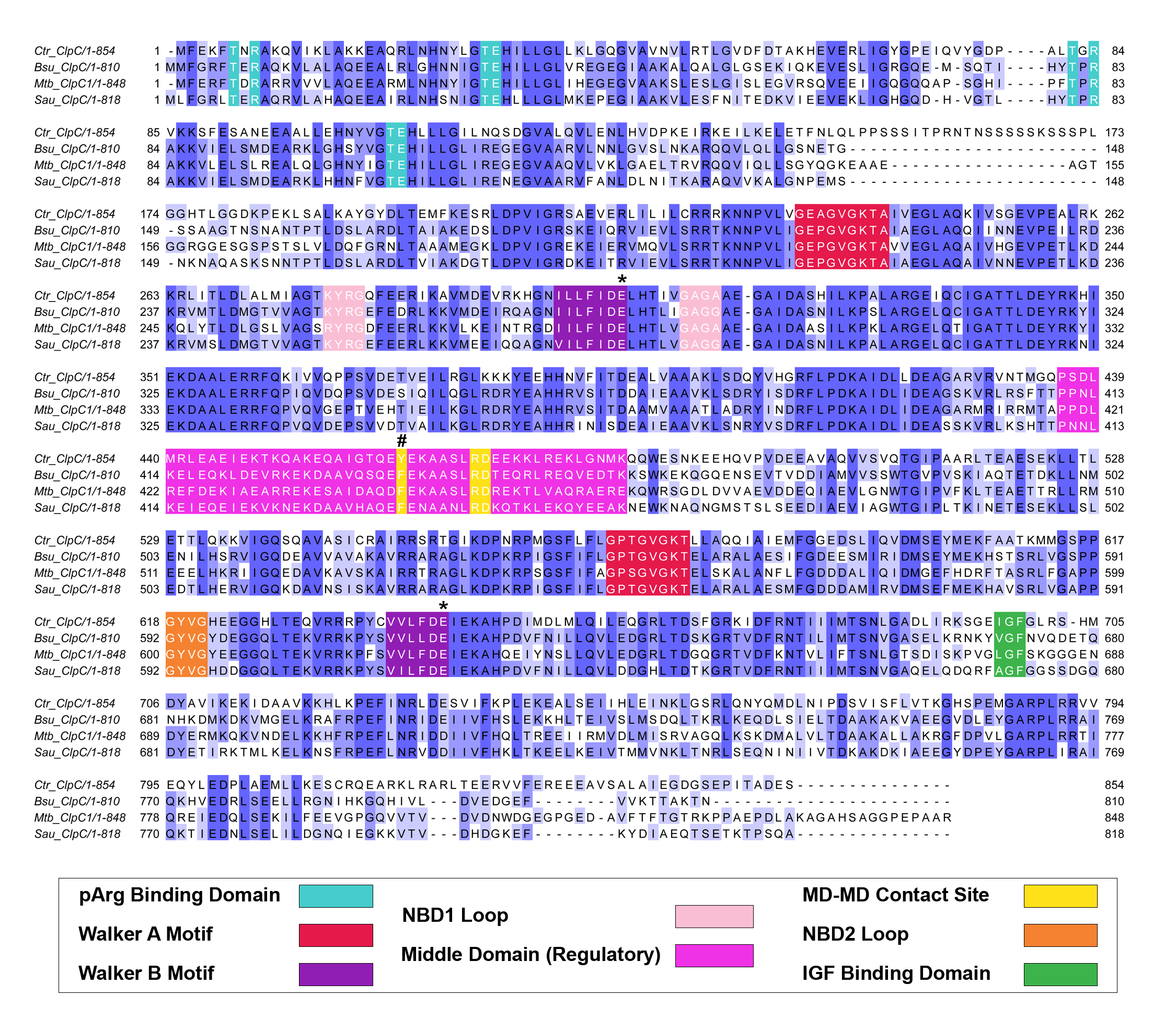

Supplement: FIG S1 [file mbio.00075-23-s0001.png]

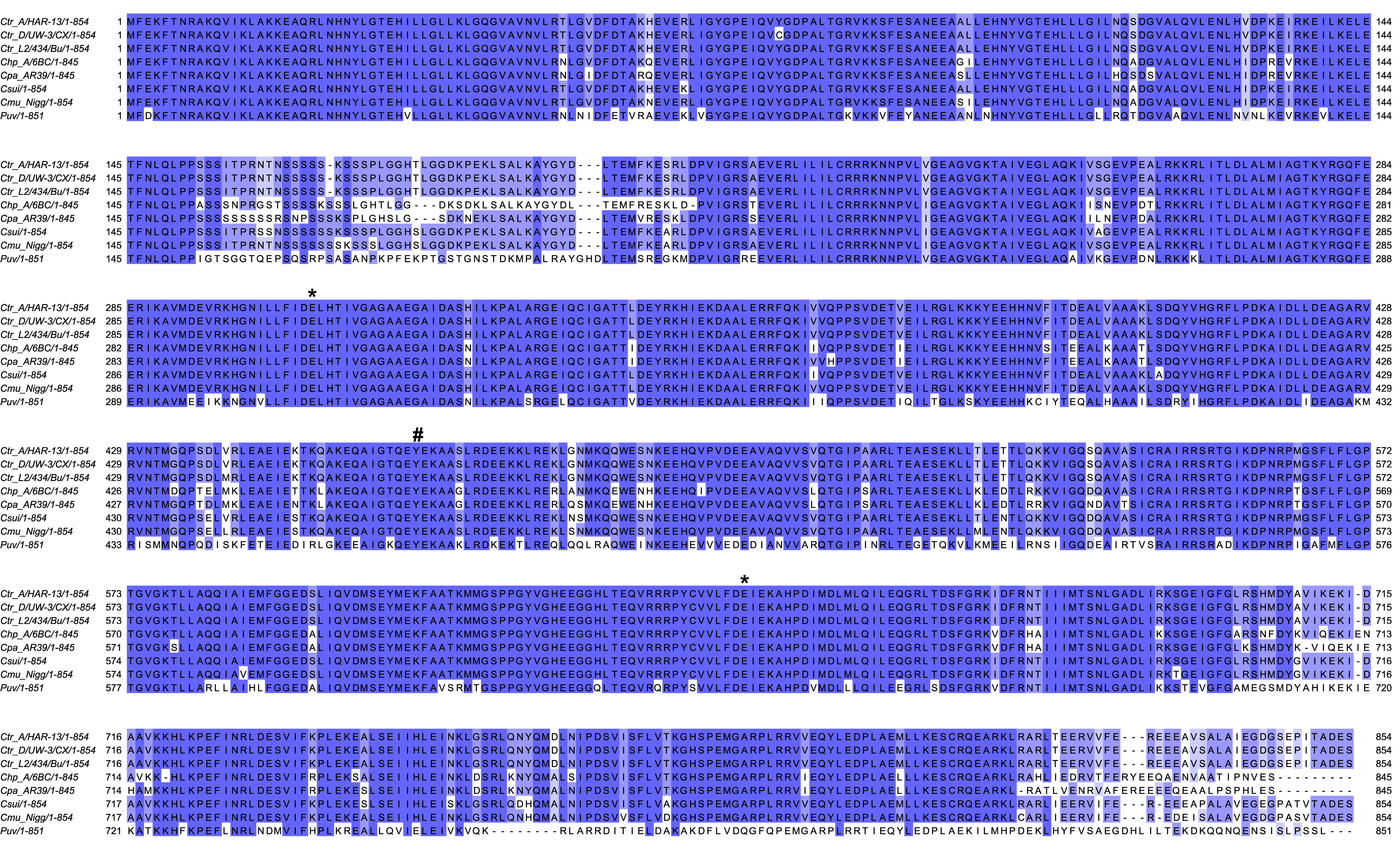

Supplement: FIG S2 [file mbio.00075-23-s0002.png]

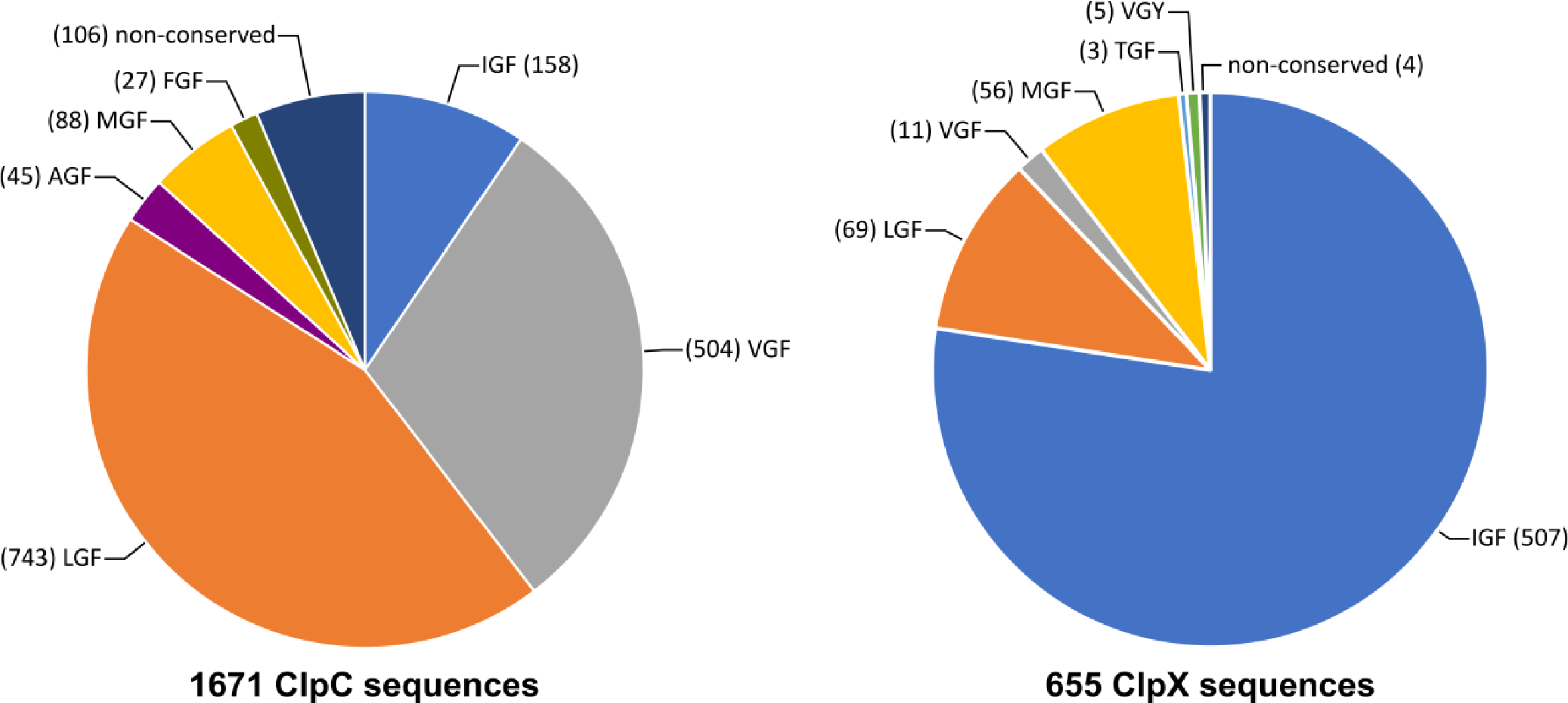

Supplement: FIG S3 [file mbio.00075-23-s0003.png]

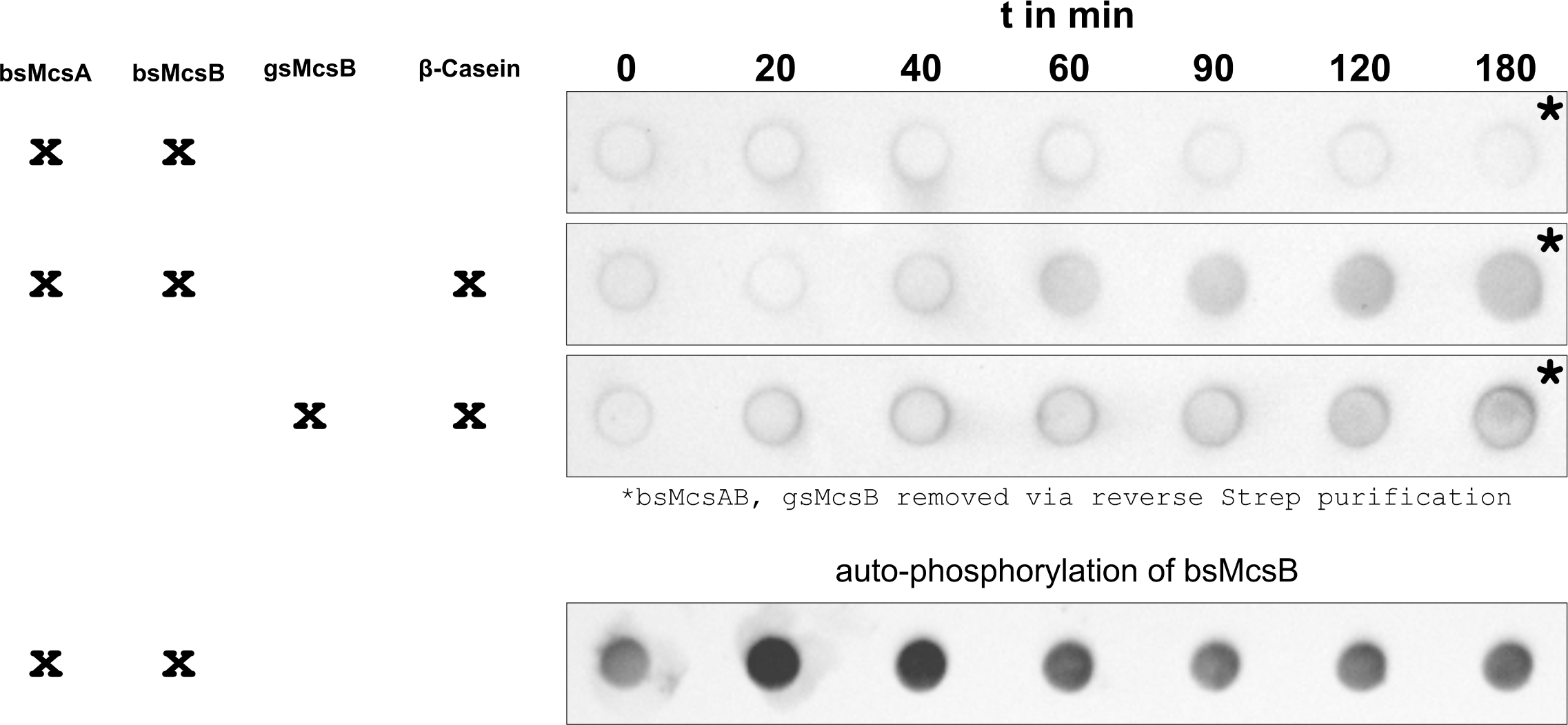

Supplement: FIG S4 [file mbio.00075-23-s0004.png]

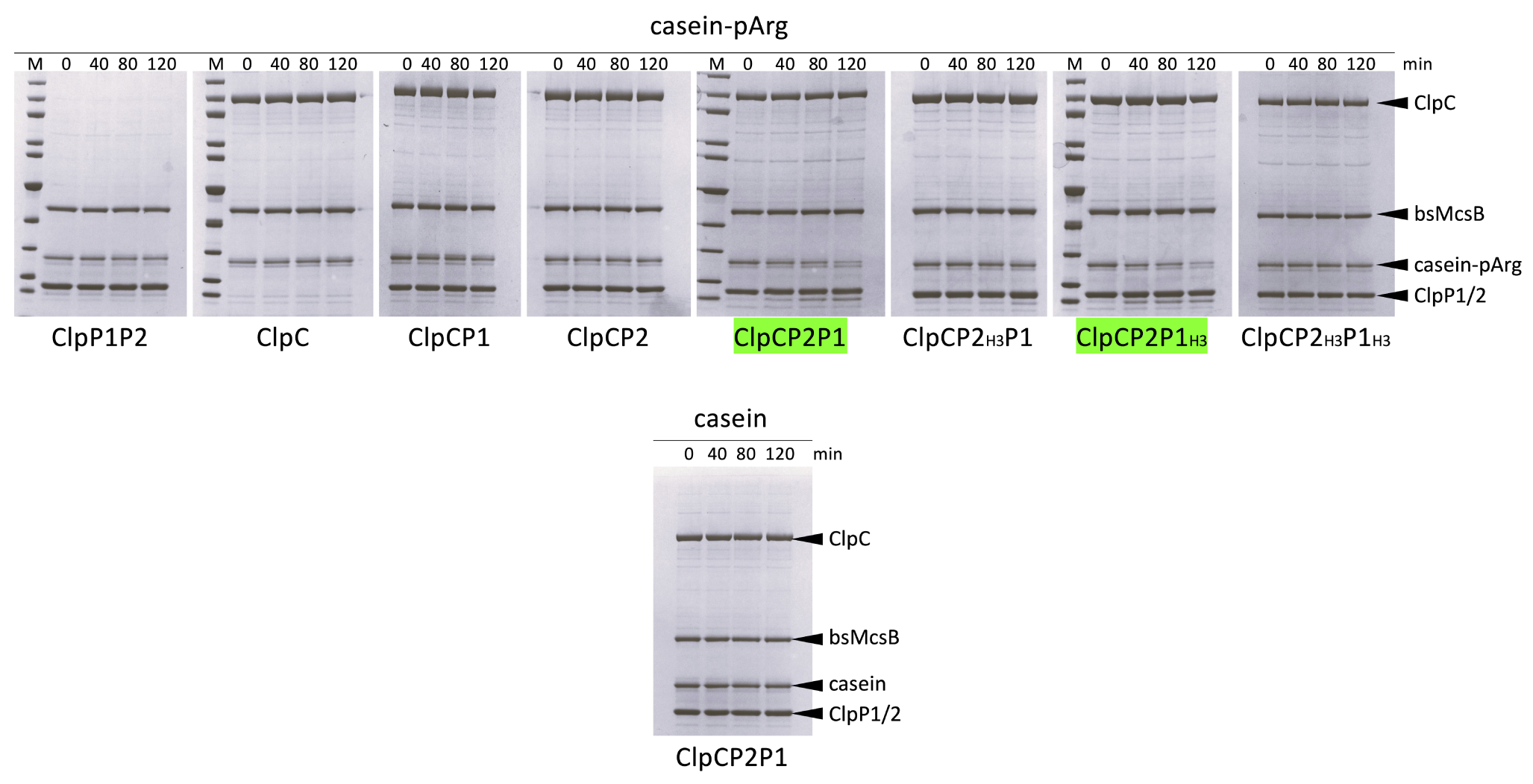

Supplement: FIG S5 [file mbio.00075-23-s0005.png]

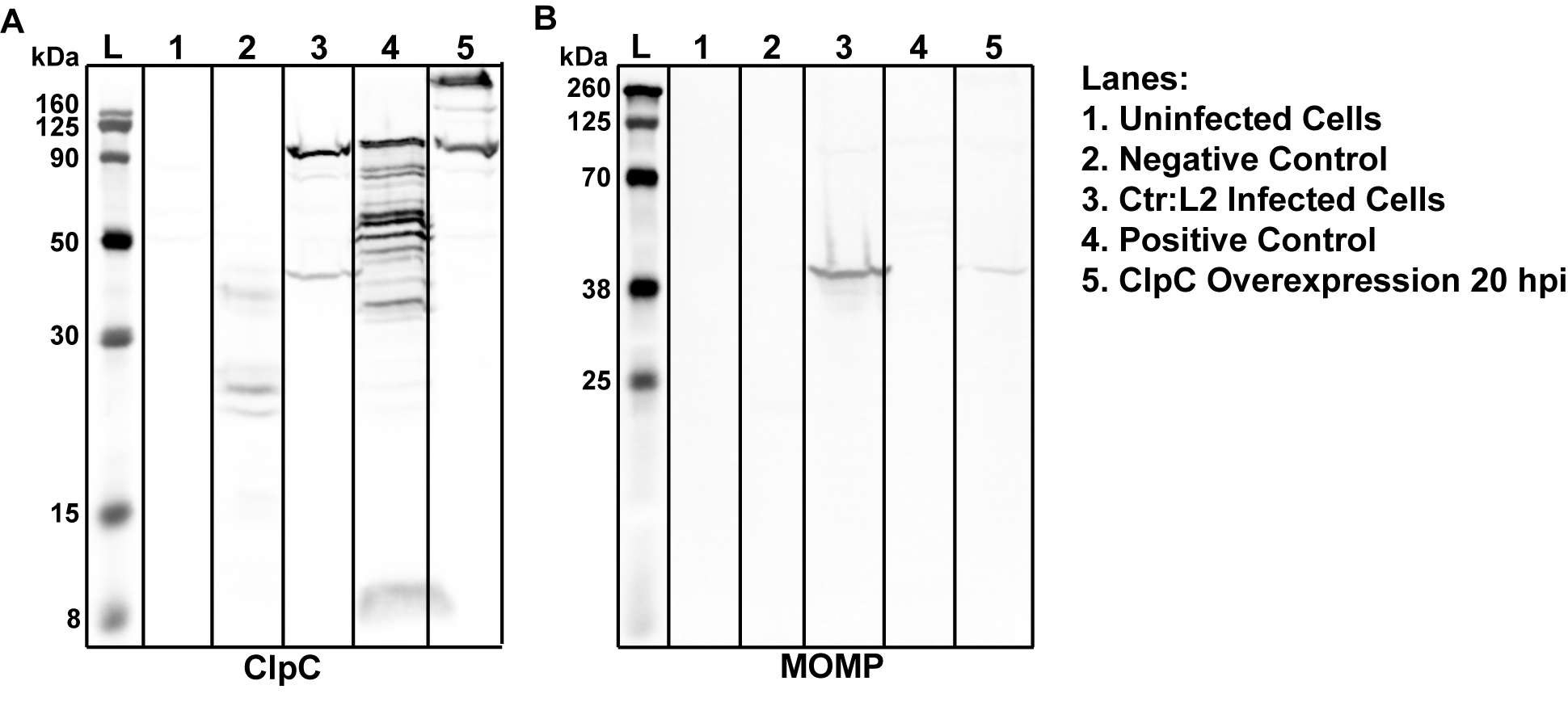

Supplement: FIG S6 [file mbio.00075-23-s0006.png]

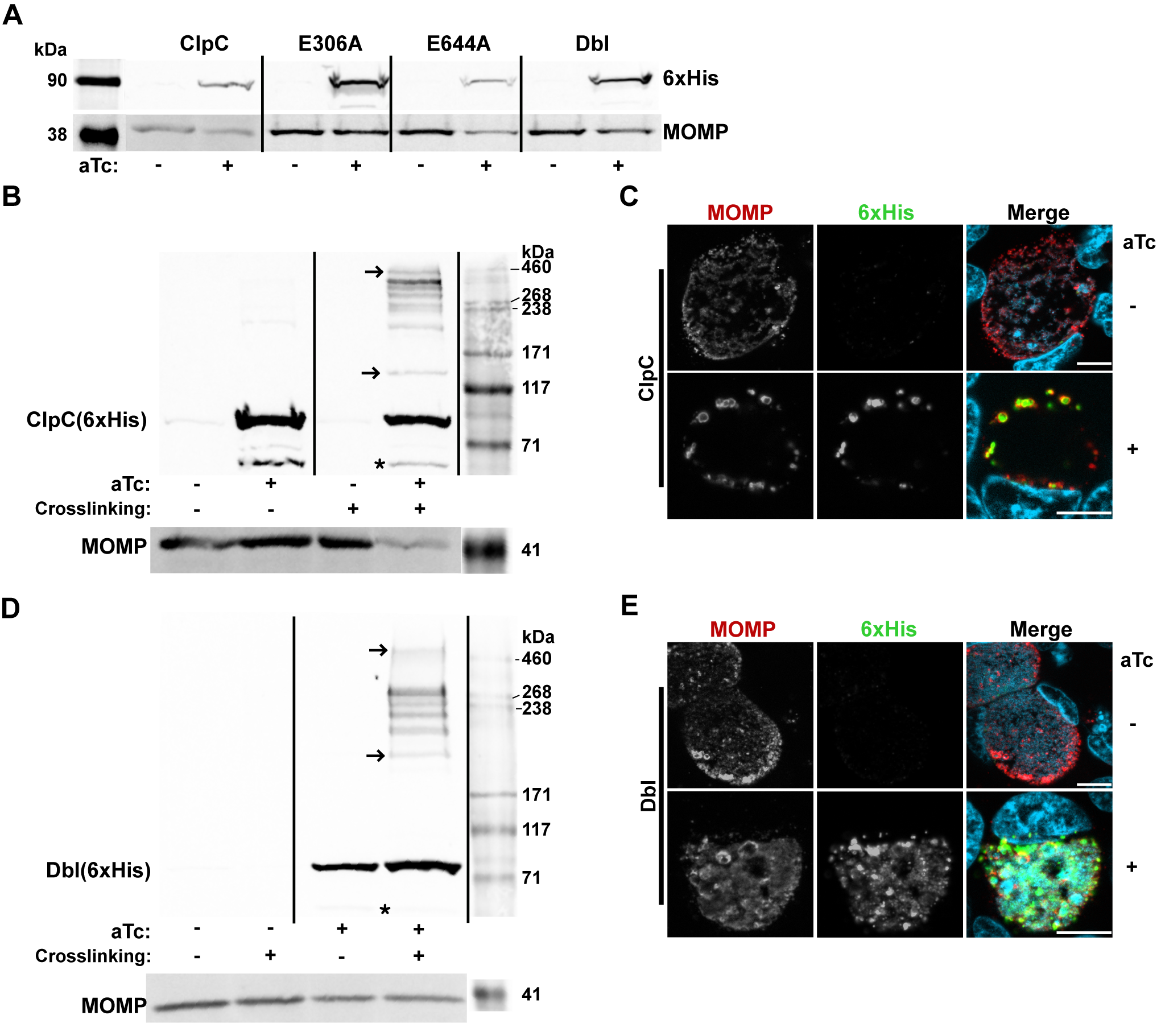

Supplement: FIG S7 [file mbio.00075-23-s0007.png]

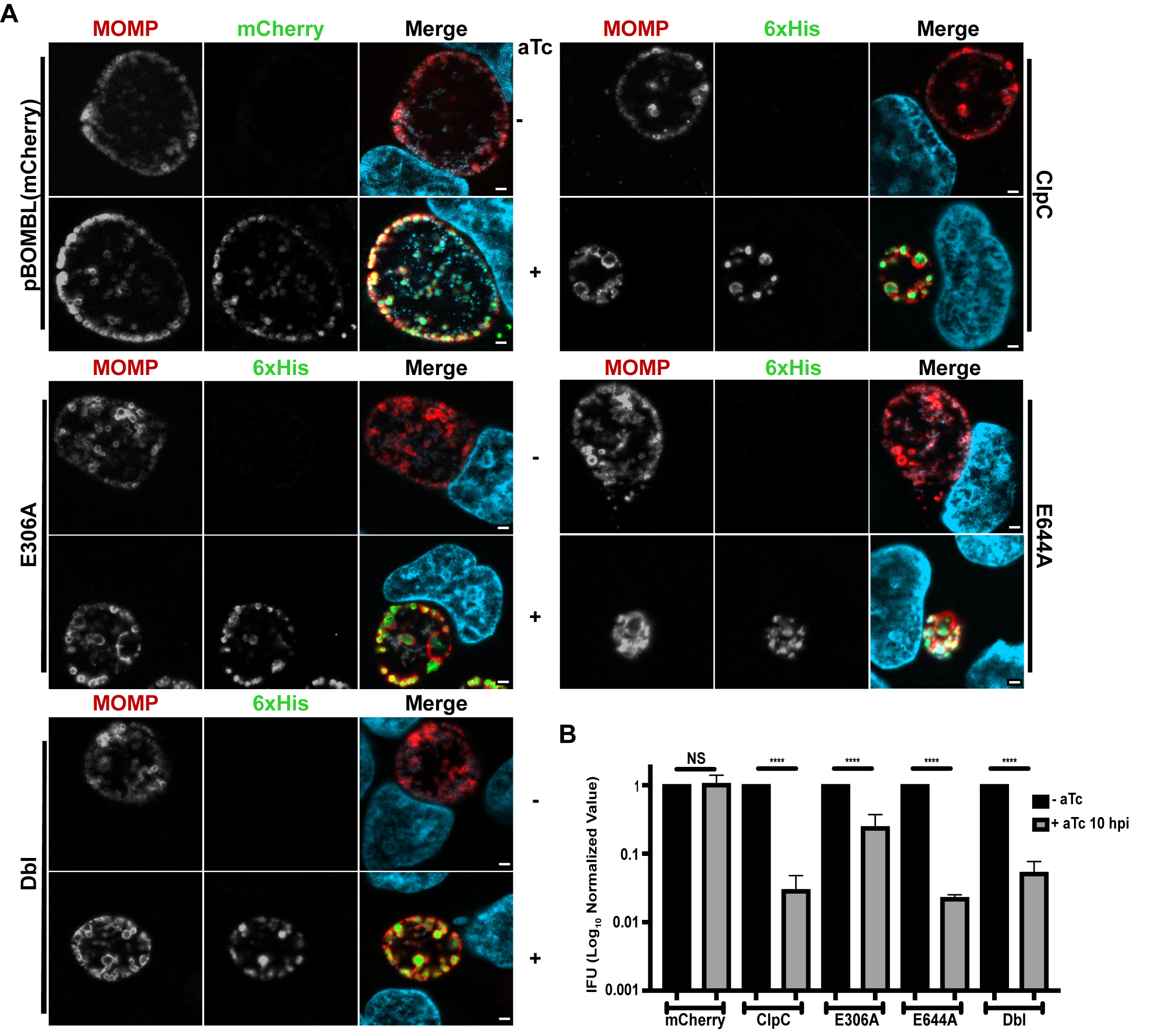

Supplement: FIG S8 [file mbio.00075-23-s0008.png]

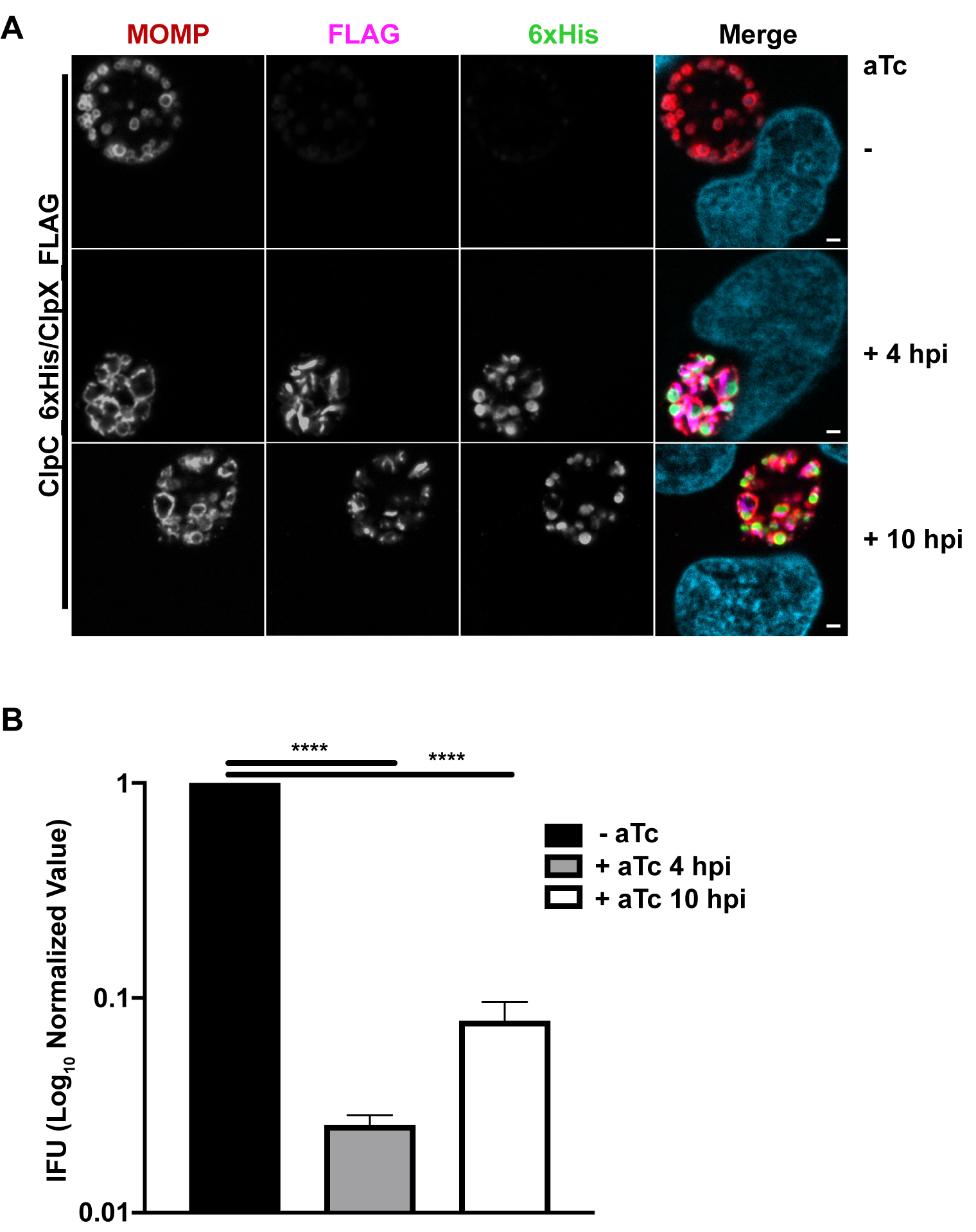

Supplement: FIG S9 [file mbio.00075-23-s0009.png]
