# Supplementary material for: Molecular Characterization of the ClpC AAA+ ATPase in the Biology of Chlamydia trachomatis
Source: mBio. 2023 Mar 28;14(2):e00075-23. doi: 10.1128/mbio.00075-23 (PMC10128030; doi:10.1128/mbio.00075-23)
Supplement: TABLE S1 [file mbio.00075-23-s0010.pdf]

Supplemental table 1: List of Plasmids, Strains, and Primers

Plasmid	Relevant genotype	Origin	Source of reference
pET-11a-*clpP1strep*	*bla* P_{T7} ::*ctclpP1strep*	pET-11a	[32]
pET-11a-*clpP2strep*	*bla* P_{T7} ::*ctclpP2strep*	pET-11a	[32]
pET-11a-*clpP1strep*$_{H3}$	*bla* P_{T7} ::*ctclpP1strep*$_{V57A,\ M77A,\ L186T}$	pET-11a	This study
pET-11a-*clpP2strep*$_{H3}$	*bla* P_{T7} ::*ctclpP2strep*$_{F63A,\ F83A,\ I190T}$	pET-11a	This study
pET-GA-*clpCstrep*	*bla* P_{T7} ::*clpCstrep*	pET-11a	This study
pET-GA-*clpCstrep*$_{E306A}$	*bla* P_{T7} ::*clpCstrep*$_{E306A}$	pET-11a	This study
pET-GA-*clpCstrep*$_{E644A}$	*bla* P_{T7} ::*clpCstrep*$_{E644A}$	pET-11a	This study
pET-GA-*clpCstrep*$_{Dbl}$	*bla* P_{T7} ::*clpCstrep*$_{E306A,\ E644A}$	pET-11a	This study
pET-MP3-*bsclpCstrep*	*bla* P_{T7} ::*bsclpCstrep*	pET-MP3	This study
pET-MP3-*mecAstrep*	*bla* P_{T7} ::*mecAstrep*	pET-MP3	This study
pET-MP3-*bsmcsAstrep*	*bla* P_{T7} ::*bsmcsAstrep*	pET-MP3	This study
pET-MP3-*bsmcsBstrep*	*bla* P_{T7} ::*bsMcsBstrep*	pET-MP3	This study
pET-MP3-*gsmcsBstrep*	*bla* P_{T7} ::*gsmcsBstrep*	pET-MP3	This study
pKT25-*clpC*	*aph* P_{lac}::*t25-clpC*	p15A	This study
pKT25-*clpC*$_{E306A}$	*aph* P_{lac}::*t25-clpC*$_{E306A}$	p15A	This study
pKT25-*clpC*$_{E644A}$	*aph* P_{lac}::*t25-clpC*$_{E306A}$	p15A	This study
pKT25-*clpC*$_{Dbl}$	*aph* P_{lac}::*t25-clpC*$_{E306A,\ E644A}$	p15A	This study
pUT18C-*clpC*	*bla* P_{lac}::*t18-clpC*	ColE1	This study
pUT18C-*clpC*$_{E306A}$	*bla* P_{lac}::*t18-clpC*$_{E306A}$	ColE1	This study
pUT18C-*clpC*$_{E644A}$	*bla* P_{lac}::*t18-clpC*$_{E644A}$	ColE1	This study
pUT18C-*clpC*$_{Dbl}$	*bla* P_{lac}::*t18-clpC*$_{E306A,\ E644A}$	ColE1	This study
pKT25-*zip*	*aph* P_{lac}::*t25-zip*	p15A	[84]
pUT18C-*zip*	*bla* P_{lac}::*t18-zip*	ColE1	[84]
pST25-*clpX*	*aadA* P_{lac}::*t25-attB1-clpX-attB2*	p15A	[30]
pUT18C-*clpX*	*bla* P_{lac}::*t18-attB1-clpX-attB2*	ColE1	[30]
pUT18C	*bla* P_{lac}::*t18*	ColE1	[84]
pBOMBL::L2	*bla* P_{Nm}::*gfp* P_{tet}::*mCherry*	pUC19	This study
pBOMBL(*clpC_6xH*)::L2	*bla* P_{Nm}::*gfp* P_{tet}::*clpC_6xH*	pUC19	This study
pBOMBL(*clpC*$_{E306A}$*_6xH*)::L2	*bla* P_{Nm}::*gfp* P_{tet}::*clpC*$_{E306A}$*_6xH*	pUC19	This study
pBOMBL(*clpC*$_{E644A}$*_6xH*)::L2	*bla* P_{Nm}::*gfp* P_{tet}::*clpC*$_{E644A}$*_6xH*	pUC19	This study
pBOMBL(*clpC*$_{Dbl}$*_6xH*)::L2	*bla* P_{Nm}::*gfp* P_{tet}::*clpC*$_{E306A,\ E644A}$*_6xH*	pUC19	This study
pBOMBL(*clpC*_6xH-*clpX_FLAG*)::L2	*bla* P_{Nm}::*gfp* P_{tet}::*clpC*_6xH/*clpX*_FLAG	pUC19	This study
pBOMBL12CRia(e.v.)::L2	*bla* P_{Nm}::*gfp* P_{tet}::As_dCas12vaa	pUC19	[50]
pBOMBL12CRia(*clpC)*::L2	*bla* P_{Nm}::*gfp* P_{tet}::As_dCas12vaa $P_{dnaKmut}$::As_crRNA_*clpC*	pUC19	This study

E. coli strain	Relevant genotype	Source of reference
DH5α	*fhuA2 Δ(argF-lacZ)U169 phoA glnV44 Φ80 Δ(lacZ)M15 gyrA96 recA1 relA1 endA1 thi-1 hsdR17*	New England BioLabs
DHT1	F^- *glnV44* (AS) *recA1 endA1 gyrA96* (Nal^R) *thi-1 hsdR17 spoT1 rfbD1 cya-854 ilv-691 ::Tn10 (TetR)*	[84]
DH10β	*Δ(ara-leu) 7697 araD139 fhuA ΔlacX74 galK16 galE15 ϕ80dlacZΔM15 (e14-) recA1 relA1 endA1 nupG rpsL* (Str^R) *rph spoT1 Δ(mrr-hsdRMS-mcrBC)*	New England BioLabs
dAPX-1	*fhuA2, [lon], ompT, gal, [dcm], ΔhsdS, λ DE3 (λ sBamHIo ΔEcoRI-B int::(lacI::PlacUV5::T7 gene1) i21 Δnin5)* Δ*clpX clpP::cam clpA::kan*	[32]

Primer name	Sequence	Features	Usage
*bsmecA*strep F	atgggtctcaggccaaATGGAAATTGAAAGAATTAACGAGCATAC	lower case for plasmid overlap construction	for amplification of *bsmecA* into pET-MP3
*bsmecA*strep R	atgggtctcagcgctTGATGCAAAGTGTTTTTTTATCGTTTCTAG	lower case for plasmid overlap construction	for amplification of *bsmecA* into pET-MP3
*bsmcsA*strep F	atgggtctcaggccaaTTGATTTGTCAAGAGTGCCACG	lower case for plasmid overlap construction	for amplification of *bsmcsA* into pET-MP3
*bsmcsA*strep R	atgggtctcagcgctCTCCTGTTCCTCCTCACTATCTG	lower case for plasmid overlap construction	for amplification of *bsmcsA* into pET-MP3
*bsmcsB*strep F	atgggtctcaggccaaATGTCGCTAAAGCATTTTATTCAGG	lower case for plasmid overlap construction	for amplification of *bsmcsB* into pET-MP3
*bsmcsB*strep R	atgggtctcagcgctTATCGATTCATCCTCCTGTCTTTTC	lower case for plasmid overlap construction	for amplification of *bsmcsB* into pET-MP3
*bsclpC*strep F	atgggtctcaggccaaATGATGTTTGGAAGATTTACAGAACG	lower case for plasmid overlap construction	for amplification of *bsclpC* into pET-MP3
*bsclpC*strep R	atgggtctcagcgctATTCGTTTTAGCAGTCGTTTTTACG	lower case for plasmid overlap construction	for amplification of *bsclpC* into pET-MP3
*gsmcsB*strep F	atgggtctcaggccaaATGTCATTTGGAAAGTTTTTCAACACAG	lower case for plasmid overlap construction	for amplification of *gsmcsB* into pET-MP3
*gsmcsB*strep R	atgggtctcagcgctTCGTTCATCACCCTCCATC	lower case for plasmid overlap construction	for amplification of *gsmcsB* into pET-MP3
GA-*ClpC*strep F	ttaagaaggagatggtaccATGTTTGAGAAGTTTACCAATCG	lower case for plasmid overlap construction	for amplification of *clpCstrep* into pET-11a via Gibson assembly

GA-*ClpC*strep R	gggttatgctaggcggccgcTTATTATTTTTCGAACTGCGGG	lower case for plasmid overlap construction	for amplification of *clpCstrep* into pET-11a via Gibson assembly
pET-GA-*clpC*strep F	gaaaaataataagcggccgcCTAGCATAACCCCTTGGG	lower case for *clpCstrep* overlap construction	for amplification of *clpCstrep* into pET-11a via Gibson assembly
pET-GA-*clpC*strep F	acttctcaaacatggtaccaTCTCCTTCTTAAAGTTAAACAAAATTATTTC	lower case for *clpCstrep* overlap construction	for amplification of *clpCstrep* into pET-11a via Gibson assembly
*clpP1*_V57A F	GGCAGCCAATTGTATTT<u>GCC</u>ATTAATAGCCCTGGAGG	upper case for V57A mutation	for mutation of hydrophobic pocket site V57 of *clpP1*
*clpP1*_V57A R	CCTCCAGGGCTATTAAT<u>GGC</u>AAATACAATTGGCTGCC	upper case for V57A mutation	for mutation of hydrophobic pocket site V57 of *clpP1*
*clpP1*_M77A F	TGCTGTTTGGGACCAAATTAAA<u>GCG</u>ATCTCTTCTCCTTTGACTACAG	upper case for V77A mutation	for mutation of hydrophobic pocket site M77 of *clpP1*
*clpP1*_M77A R	CTGTAGTCAAAGGAGAAGAGAT<u>CGC</u>TTTAATTTGGTCCCAAACAGCA	upper case for V77A mutation	for mutation of hydrophobic pocket site M77 of *clpP1*
*clpP2*_F63A F	TCCTACTAAGGATATTCAAATT<u>GCC</u>ATCAACTCTCCAGGGGGATATA	upper case for F63A mutation	for mutation of hydrophobic pocket site F63 of *clpP2*
*clpP2*_F63A R	TATATCCCCCTGGAGAGTTGAT<u>GGC</u>AATTTGAATATCCTTAGTAGGA	upper case for F63A mutation	for mutation of hydrophobic pocket site F63 of *clpP2*
*clpP2*_F83A F	CGATTTACGACACCATTCGT<u>GCC</u>CTAGGCTGTGATGTAAATAC	upper case for F83A mutation	for mutation of hydrophobic pocket site F83 of *clpP2*
*clpP2*_F83A R	GTATTTACATCACAGCCTAG<u>GGC</u>ACGAATGGTGTCGTAAATCG	upper case for F83A mutation	for mutation of hydrophobic pocket site F83 of *clpP2*
*clpC*_E306A F	CCTCTTATTTATTGAT<u>GAG</u>CTTCACACGATTGTTG	upper case for E306A mutation	for mutation of the NBD1 *clpC* Walker B motif
*clpC*_E306A R	CAACAATCGTGTGAAG<u>CTC</u>ATCAATAAATAAGAGG	upper case for E306A mutation	for mutation of the NBD1 *clpC* Walker B motif
*clpC*_E644A F	TGTTGTGTTGTTTGAT<u>GAA</u>ATTGAAAAAGCACATC	upper case for E644A mutation	for mutation of the NBD2 *clpC* Walker B motif
*clpC*_E644A R	GATGTGCTTTTTCAAT<u>TTC</u>ATCAAACAACACAACA	upper case for E644A mutation	for mutation of the NBD2 *clpC* Walker B motif

pLATE31 clpC F	agaaggagatataactATGTTTGAGAAGTTTACCAATCGCGCAAAGCAAG	lower case for plasmid overlap construction	for amplification of *clpC* into pLATE31
pLATE31 clpC R	*gtggtggtgatggtgatg*gccTGATTCATCAGCTGTAATAG	Lower case for plasmid overlap construction, italic for 6xHis within plasmid backbone	for amplification of *clpC* into pLATE31
clpC/(pKT25)/5'LIC	ctgcagggtcgactctagagTTTGAGAAGTTTACCAATCG	lower case for plasmid overlap construction	for amplification of *clpC* into pKT25
clpC/(pKT25)/3'LIC	attcttagttacttaggtacTTATGATTCATCAGCTGTAATAG	lower case for plasmid overlap construction	for amplification of *clpC* into pKT25
clpC/(pUT18C)/5'LIC	actgcaggtcgactctagagTTTGAGAAGTTTACCAATCG	lower case for plasmid overlap construction	for amplification of *clpC* into pUT18C
clpC/(pUT18C)/3'LIC	gatgaattcgagctcggtacTTATGATTCATCAGCTGTAATAG	lower case for plasmid overlap construction	for amplification of *clpC* into pUT18C
clpC/(pBOMBL)/5' LIC	aaagatcttcacacaggacatctgcATGTTTGAGAAGTTTACCAATC	Lower case for plasmid overlap construction	For amplification of *clpC* and mutants into pBOMBL
clpC(6xHis)/(pBOMB/L)/3' LIC	acatatttgaatggtcgaccggtactta*atggtgatggtgatggtg*TGATTCATCAGCTGTAA	Lower case for plasmid overlap construction, italic for addition of 6xHis to *clpC*	For amplification of *clpC* and mutants with a 6xHis tag into pBOMBL
clpC/*clpX_linker*/3' LIC	tttttgtcatGCAGATTCTCCTCTTAATG	Lower case for plasmid overlap construction	For amplification of *clpC_clpX* duel expression into pBOMBL
clpX/*clpC_linker*/5' LIC	gagaatctgcATGACAAAAAAAAATCTTGCGGTCTGTTCTTTTTGTGGACGATCTGAG	Lower case for plasmid overlap construction	For amplification of *clpC_clpX* duel expression into pBOMBL
clpX/(pBOMBL)/3' LIC	tttgaatggtcgaccggtacTTA*TTTGTCGTCATCGTCCTTATAATC*AGCAATCGCCTCTGGTGATT	Lower case for plasmid overlap construction, italic for addition of FLAG to *clpX*	For amplification of *clpC_clpX* with a FLAG tag into pBOMBL
clpC/mutE306A/Q5_F	ATTTATTGAT<u>GCA</u>CTTCACACGATTGTTG	Underline for E306A mutation	for mutation of the NBD1 *clpC* Walker B motif
clpC/mutE306A/Q5_R	AAGAGGATGTTCCCATGTTTAC	For E306A mutagenesis	for mutation of the NBD1 *clpC* Walker B motif

clpC/mutE644A/Q5_F	GTTGTTTGATGCTATTGAAAAAGCACATCC	Underline for E644A mutation	for mutation of the NBD2 *clpC* Walker B motif
clpC/mutE644A/Q5_R	ACAACACAGTAAGGGCGG	For E644A mutagenesis	for mutation of the NBD2 *clpC* Walker B motif
ct286 *clpC* qPCR F	CTCTTCCTCCATCACTCCTAGA	Forward qPCR primer	For qPCR of *clpC*
ct286 *clpC* qPCR R	AGGTTTATCTCCGCCCAAAG	Reverse qPCR primer	For qPCR of *clpC*
ct446 *euo* qPCR F	CGAAGACTACTCGTTGGGAAATA	Forward qPCR primer	For qPCR of *euo*
ct446 *euo* qPCR R	AACAGAAGCTCTCCTTGATAAGT	Reverse qPCR primer	For qPCR of *euo*
ct119 *incA* qPCR F	TCTGATCGCTCCACAAATCAC	Forward qPCR primer	For qPCR of *incA*
ct119 *incA* qPCR R	CTTCTCTTTGCAGATCCTGGTATA	Reverse qPCR primer	For qPCR of *incA*
ct431 *clpP1* qPCR F	GATGCTGGGTTTGCTGTTTG	Forward qPCR primer	For qPCR of *clpP1*
ct431 *clpP1* qPCR F	CAGATCCCATAGATGCTGCTAAA	Reverse qPCR primer	For qPCR of *clpP1*

**gBlock Name**	**Sequence**	**Features**	**Usage**
clpC crRNA	tgtgaaagtgggtcttaagacgtcggtactgcatgtgacgcacgtagatcatgca*TTCACCGGTGGAGACGGTTTTCTTATAATGACACC*TAATTTCTACTCTTGTAGAT**CCAATCGCGCAAAGCAAGTGA**CAAATAAAACGAAAGGCTCAGTCGAAAGACTGGGCCTTTCGTTTTATcaacagcggtctactgaatctgagctagtgcgtgatataattaaaattatattca	Lower case for plasmid overlap and spacer, *italicized* for $P_{dnaKmut}$ promoter sequence, underlined for crRNA scaffold, **bold** for *clpC* targeting sequence, Upper case for rrnB1 terminator	For CRISPRi knockdown of *clpC*
